# Health care cost accounting in the Indian hospital sector

**DOI:** 10.1093/heapol/czae040

**Published:** 2024-05-30

**Authors:** Yashika Chugh, Shuchita Sharma, Abha Mehndiratta, Deepshikha Sharma, Basant Garg, Shankar Prinja, Lorna Guinness

**Affiliations:** Department of Community Medicine and School of Public Health, Postgraduate Institute of Medical Education and Research, Sector 12, Chandigarh 160012, India; Department of Community Medicine and School of Public Health, Postgraduate Institute of Medical Education and Research, Sector 12, Chandigarh 160012, India; Global Health Policy Program, Center for Global Development, Europe, Great College St, London SW1P 3SE, United Kingdom; Department of Community Medicine and School of Public Health, Postgraduate Institute of Medical Education and Research, Sector 12, Chandigarh 160012, India; Government of India, National Health Authority, Tower-L, Jeevan Bharti, Janpath, Connaught Place, New Delhi 110001, India; Department of Community Medicine and School of Public Health, Postgraduate Institute of Medical Education and Research, Sector 12, Chandigarh 160012, India; Global Health Policy Program, Center for Global Development, Europe, Great College St, London SW1P 3SE, United Kingdom

**Keywords:** Cost accounting, hospital cost accounting, financial accounting, cost reporting, price setting in health, India, health insurance reimbursement

## Abstract

Setting reimbursement rates in national insurance schemes requires robust cost data. Collecting provider-generated cost accounting information is a potential mechanism for improving the cost evidence. To inform strategies for obtaining cost data to set reimbursement rates, this analysis aims to describe the role of cost accounting in public and private health sectors in India and describe the importance, perceived barriers and facilitators to improving cost accounting systems. In-depth interviews were conducted with 11 key informants. The interview tool guide was informed by a review of published and grey literature and government websites. The interviews were recorded as both audio and video and transcribed. A thematic coding framework was developed for the analysis. Multiple discussions were held to add, delete, classify or merge the themes. The themes identified were as follows: the status of cost accounting in the Indian hospital sector, legal and regulatory requirements for cost reporting, challenges to implementing cost accounting and recommendations for improving cost reporting by health care providers. The findings indicate that the sector lacks maturity in cost accounting due to a lack of understanding of its benefits, limited capacity and weak enforcement of cost reporting regulations. Providers recognize the value of cost analysis for investment decisions but have mixed opinions on the willingness to gather and report cost information, citing resource constraints and a lack of trust in payers. Additionally, heterogeneity among providers will require tailored approaches in developing cost accounting reporting frameworks and regulations. Health care cost accounting systems in India are rudimentary with a few exceptions, raising questions about how to source these data sustainably. Strengthening cost accounting systems in India will require standardized data formats, integrated into existing data management systems, that both meet the needs of policy makers and are acceptable to hospital providers.

Key Messages

- Collecting provider-generated cost accounting information will be a key mechanism for improving the cost evidence to inform reimbursement rates or tariffs for public health insurance schemes in India.
- Providers recognize the value of cost analysis but have mixed opinions on gathering and report cost information citing resource constraints and a lack of trust in payers.
- Strengthening cost accounting systems in India will require standardized data formats, integrated into existing data management systems, that both meet the needs of policy makers and are acceptable to hospital providers.

## Introduction

In 2018, the Government of India launched the flagship Ayushman Bharat scheme as part of its strategy for universal health coverage (UHC) (Sarwal *et al*., 2021). Ayushman Bharat encompasses the world’s largest tax-funded health assurance scheme Pradhan Mantri Jan Arogya Yojana (PM-JAY) (National Health Authority, 2019), providing the poor and vulnerable with curative care services. The potential of the PM-JAY platform to achieve its objectives will, in part, be contingent upon setting priorities and reimbursement tariffs that ensure widespread health care provider participation while obtaining the best value for the available budget. In turn, this will rely on the availability of robust evidence on disease burden, service availability and use and costs.

Implementation of PM-JAY is entrusted to the National Health Authority (NHA) and—as part of a transition from volume-based to value-based care—the hospitals empanelled with PM-JAY are being paid using case-based bundled payments (National Health Authority, 2019). The NHA sets the reimbursement rates for and regularly updates the list of health benefits packages (HBP). The setting of reimbursement rates is currently informed by evidence from a one off nationally representative costing survey, evidence from economic evaluations and expert consultation (Prinja *et al*., 2021; Chauhan *et al*., 2022; National Health Authority, Government of India, New Delhi, 2022). While the use of evidence on health care costs is critical, costing exercises are resource and time intensive and have rapidly become outdated. At the same time, empanelled providers complain of the inadequacy of the reimbursement rates (Federation of Indian Chambers of Commerce and Industry (FICCI), 2018) and the flat rate payment that does not adjust for severity and complexity. These issues raise the risk of providers declining to participate in the scheme, balance billing or selectively denying hospitalization to sicker and more vulnerable patients.

The NHA is considering reforms to the payment scheme, similar to a diagnosis-related group-based payment mechanism, built on a classification system which factors in the patient case mix and severity. Such systems rely on transparent and robust evidence to inform both diagnosis groupings and their reimbursement tariffs. In particular, data are needed for standardized patient classification around diagnosis and procedures, how costs might vary with case mix and severity and the average costs of providing health services. These data could be generated through a sustainable national cost system that produces reliable time-relevant cost information based on standardized costing methods (Barber *et al*., 2019). However, the scope to capture these data at a national level in this way in India is unclear.

National health care costing systems found in many countries are fundamental for informing the design of case-based payment schemes (e.g. England, Australia, Germany and Thailand) (Tan *et al*., 2014; Patcharanarumol *et al*., 2018; Barber *et al*., 2019; Australian Hospital Patient Costing Standards, 2023; National Cost Collection for the NHS, 2022). By accurately capturing direct and indirect costs associated with procedures, cost systems enable health care organizations to make informed decisions regarding reimbursements, resource allocation and process optimization. Incorporating cost information into rate-setting processes also enhances credibility and transparency, providing stakeholders with a clear understanding of the rationale behind pricing decisions. Moreover, establishing a direct link between costs and reimbursement rates enables policy-makers and the public to assess the efficiency and effectiveness of health care spending. This transparency fosters trust and accountability, enhancing the overall stewardship of public resources in health care.

National costing systems rely on various different costing methods. The gold standard method used for national reference costs is cost accounting (e.g. England and Australia) (National Cost Collection for the NHS, 2022). Cost accounting determines the cost of each service or product (Chapman *et al*., 2016); explains the production processes, how this links to costs; and provides detailed information for use in analytics around understanding the level of efficiency, the quality of care and, when linked to health outcomes, the value of care (Kaplan and Witkowski, 2014; Malmmose and Lydersen, 2021). At the hospital level, cost accounting is vital for the accurate measurement of costs and therefore financial and managerial performance (The Institute of Cost Accountants of India, 2015; Carroll and Lord, 2016; Soni, 2023). Cost accounting methods have evolved over time (Kaplan and Porter, 2011) and include multiple techniques that respond to different needs, varying in their levels of granularity, complexity and data requirements (see Supplementary Annexure 1 for a summary of the most commonly used methods) (Selto and Widener, 2004; Bonner *et al*., 2012; Carroll and Lord, 2016).

Cost systems should be tailored to the context and data availability, while also allowing for adaptability and investment in complexity as data infrastructure improves. As a result, national costing systems range from sporadic costing studies to routine national surveillance efforts (Raulinajtys-Grzybek, 2014; Bredenkamp *et al*., 2020). The diversity arises from decisions regarding data collection processes, the stage of reimbursement system development, regulatory frameworks around cost accounting and the chosen costing methodologies (Guinness *et al*., 2022). In some instances, comprehensive cost accounting surveys involve all participating providers, as mandated in the UK and Australia, while in others, only representative samples of providers are utilized, such as in France, Germany and Thailand (Barber *et al*., 2019). Other countries require all providers to submit data but use charge/billing data as a proxy for costs (e.g. USA, Taiwan) although this can result in aligning rates with provider’s profit incentives rather than strategic priorities of the national purchasing agency (Tan *et al*., 2014). In settings where there are limiting factors such as budget constraints, mixed health systems, poor data and weak regulation, simpler methods like utilizing expenditure data or gathering information from smaller facility samples have been employed for price setting. Alternative approaches, exemplified in India, Cambodia and Kenya, involve implementing baseline multisite costing studies to kick-start the process (Mathauer, 2011; Jacobs *et al*., 2019; Prinja *et al*., 2020), providing foundational evidence, best practices or pilots for future development. Experiences in Thailand, Kyrgyzstan and China showcase how cost systems can evolve iteratively from initial one-time exercises into complex systems with increasing provider participation as capacity evolves (Mathauer and Wittenbecher, 2013; Bredenkamp *et al*., 2020).

A national-level sustainable cost system based on provider-level cost accounting systems could be used to inform the HBP review process for India as well as address other national costing needs such as reference costs, efficiency analysis and cost evidence for health technology assessment. However, it is vital to first understand the current state of how cost information is maintained in the hospital sector to understand the capacity for generating the required data. This analysis aims to develop an understanding of the role of cost accounting in public and private health sectors in India to inform strategies for improving the cost evidence base for the setting of reimbursement rates.

## Methods

### Study design

We employed a qualitative study design where in-depth interviews (IDIs) with individuals were conducted to enable attention to the context and processes and for participants to describe their views and experiences in detail. The respondents for IDIs were chosen to include experts in the area of health care cost accounting in India, key individuals (academics or cost accountants) or representatives of organizations that have been involved in hospital costing and individuals who could represent the views of hospital networks/associations and industry bodies. To further substantiate the findings, a virtual panel consisting of 32 experts from government agencies, non-governmental organizations, private sector, health care industry partners and health insurance industry was conducted (Supplementary Annexure 2). The panels were requested to reflect and feedback on the initial findings from the IDIs and provide further reflections and perspectives on the key study questions (Supplementary Annexe 5).

### Sampling and recruitment

For the qualitative interviews, we identified potential participants through various routes. Firstly, a rigorous review of grey and peer-reviewed hospital costing literature in India was identified through consultation within the research team, public health databases (PubMed) and internet searching. The following search strategy was deployed: ‘((((((((healthcare) OR (hospitals)) OR (healthcare management)) OR (hospital operations)) AND (healthcare cost accounting)) OR (healthcare costs)) OR (cost accounting)) AND (India))’. The review process was supplemented by a review of the websites of various government bodies leading us to reports of cost accounting–related work done by government bodies and different researchers working on cost accounting in health in India. We compiled a list of committee members and authors for those reports and papers. Next, we listed the key organizations that represent health care providers in India. We sought to identify and contact individuals identified in the document review as well as representatives from the key health care provider organizations who were willing to discuss the role of cost accounting in India. To supplement this, we also approached a few key respondents based on personal contacts of those in our research and policy network, looking at websites and snowballing with individuals interviewed to suggest others. Having identified potential participants, we contacted 12 individuals to obtain maximum diversity within our sample according to their roles, professional backgrounds and level of experience. Participants for the panel discussion were selected through the key informants and information generated by the IDIs. In addition, we also invited representatives from and those suggested by the National Health Authority, which is the apex body for Ayushman Bharat—Pradhan Mantri Jan Arogya Yojana, India’s flagship health insurance programme.

### Data collection

The potential respondents were approached by email describing the broad aims and objectives of the study as well as expectations from the interview. A concept note of the study was also circulated if further details were sought by the interviewee. The interviewees were assured that they would not be named or identifiable in any way. Given the dispersed nature of the interviewees and research team, we interviewed the participants virtually. Written and verbal consent was sought to record the interviews. The authors made reflective notes to assist with the analysis. All the interview audios/videos and transcripts were given codes to maintain anonymity in the final write up, the access to which was limited to the authors.

### Interview tool development and overview of themes

**Figure 1.**
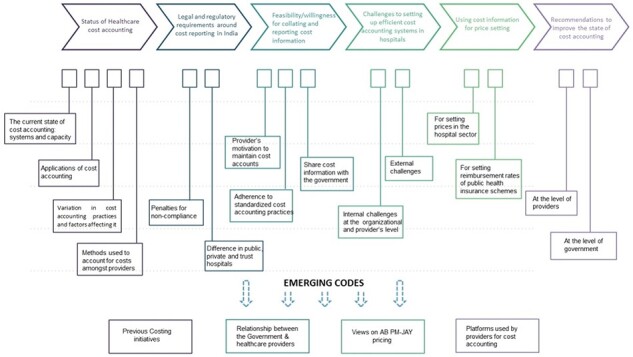
Representation of the a priori and emergent codes extracted from the interviews

An exhaustive interview tool guide (Supplementary Annexure 3) was developed and piloted to ensure that key areas were covered. The tool guide was informed by the review of literature on the role of cost accounting in the health care industry and the need for a cost data collection system in India, keeping in view the overall aim of the qualitative study. The key themes included in the guide were aimed at understanding the existing state of cost accounting in the hospital sector in India, the legal requirements around reporting of health care cost accounts, motivation and barriers to setting up and implementing cost accounting systems, price setting in the hospital sector including that for publicly financed health insurance schemes, willingness to report cost accounts to any central government organization and recommendations for improving cost accounting practices in hospitals in India (Figure 1).

### Data analysis

**Figure 2.**
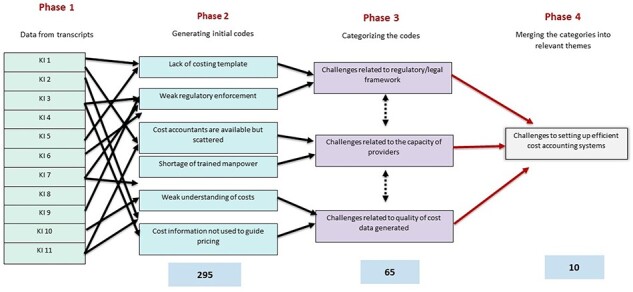
Coding framework for analysis

All interviews were recorded for both audio and video. A verbatim transcript was developed, checked and anonymized. The data were analysed thematically to make comparisons within and across the interviews, also allowing us to capture specific issues discussed by the interviewees. To do this, a coding frame was drafted and refined. The authors independently read all the transcripts to provide codes in response to the different sections in the transcript, followed by merging their codes to eventually group the themes as a priori and emergent themes (Stemler, 2000). Finally, the framework method was applied to the interview transcripts conducted at different times in the data collection process (Goldsmith, 2021). Multiple discussions were held to add, delete, classify or merge the existing themes (Figure 2). The themes and related excerpts from all the interviews were collated for analysis in MS Excel to allow for comparison of findings and reflection on differences (Supplementary Annexure 4). Once the initial findings were collated, they were presented to the costing panel for validation whose feedback was incorporated into the thematic analysis using the coding framework (Figure 2) to segregate information. The relevant information was then added to the text in the manuscript under relevant themes.

## Results

### Participants’ characteristics

A total of 12 participants were invited to interview, including a mix of those belonging to various health care provider organizations in India from the government, private and not-for-profit sectors as well as from the academia. Out of the 12 participants invited, semi-structured interviews were conducted with 11 participants. One did not respond to the invite (Table 1).

**Table 1.** Characteristics of the key informants interviewed for the study

Type of organization	No. of interviewees
Private Provider Chain of Hospitals	3
Industry representatives (Association of Healthcare Providers of India, Confederation of Indian Industry)	3
Academia	2
Ministry of Finance, Government of India	1
Public sector provider (Institute of Medical Sciences)	1
Insurance provider	1

### Findings

#### Status of health care cost accounting in India

The interviews explored the status of cost accounting in terms of both current practices and the capacity to implement cost accounting. The consensus from key informants (KI) is that cost accounting practices in India today are ‘more rudimentary’ (KI 1; KI 9), that the hospital industry ‘is not very mature in terms of costing systems’ (KI 5) and that ‘99% of providers … do not have any insight into their costs’ (KI 10). ‘It’s only the big players who really have done costing’ (KI 3), i.e. in the big private chain hospitals, which represent at most 10–20% of providers (Assessment of the Healthcare Delivery Market in India, 2023). Eight of the 11 participants agreed that the majority of health care providers are made up of ‘players who have absolutely no idea what is the costing’ (KI 3), and the common practice is ‘thumb rule costing [which is when] you understand with guesswork’. In addition, the interviewees (4 out of 11) felt that the limited attempts by providers to put cost accounting in place were likely because ‘they are more concerned with taking care of their hospital patients’ (KI 3) than on financial management processes. However, one key informant from the non-governmental organization (NGO) sector mentioned that they had developed their own software, which encompasses finance, electronic medical records, radiology information and management information systems and allows for the extraction of granular information. For instance: ‘So, if it is a robotic pyeloplasty, I can query it for the last 100 Pyeloplasty. What would be the consumable use? What would be the instrument use? I can retrieve that data and I understand whether by each surgeon, by the different surgeons, what has been an estimate’ (KI 8).

When reflecting on the public sector, three participants pointed out that the ‘exercise has not been done even in public institutions’ (KI 8). As public hospitals have guaranteed funding and do not need to set prices, ‘the public sector is not bothered about how much it is costing at the management level’ (KI 9). On the other hand, it was reported by one of the respondents that some tertiary care public sector hospitals have begun to include finance officers in their teams and started focusing on analysing costs.

When asked about general capacity for cost accounting in India, participants’ views were captured by this statement ‘obviously this expertise is available, but it is scattered. It is not available at one place’ (KI 1) and that the national cost accounting body had limited experts in health care. In addition, 4 out of the 11 respondents stated that ‘there was not a well-structured framework’ (KI 3, 4, 6, 7) for costing and that there is a need for the government to develop and provide a template. The respondents also raised the issues that cost accountants in the health sector ‘are not available’ (KI 8) and that the costing process is time consuming and is ‘costlier’ (KI 1). In addition to this, experts who had been involved in costing studies found capacity for costing at the hospital level limited, ‘it was a complex exercise, the hospital people did not understand’ (KI 3). Two respondents also highlighted that capturing human resource costs is very difficult as it is not standard practice to track the time spent by staff in different activities. As a result, where costs are tracked, the focus is on the estimation of direct costs only.

At the same time, interviews revealed that cost accounting procedures can and are being used although the users often do not recognize it: ‘Most of the private providers, they may not know the signs of cost accounting but they do the costing. We need to figure out what our costs are, how we can control, drive down, optimize our costs, keep prices affordable and still, you know, deliver return expectations of our investors’ (KI 1).

There have been a few costing initiatives including attempts to build a standardized costing template for use in the Indian hospital sector. One expert described the design of a tool for time-driven activity-based costing with marginal costing. Others described costing exercises carried out to inform reimbursement rates, including one by the Institute of Cost Accountants, for which a costing template was developed for health care providers. However, uncertainty exists around the reach and usability of these costing initiatives as well as how the findings were used by the government: ‘it has not worked out’ (KI 7).

#### Legal/regulatory requirements around cost reporting in India

While financial accounts provide information about profit and loss, cost accounts provide detail on the relative costs of different specified areas of expenditure or cost e.g. human resources, capital, consumables, etc. All corporations and trusts submit financial accounts as part of a tax return and, additionally, there is a regulatory requirement to submit cost accounts under corporation law and clinical establishment law (Government of India, 2014) but ‘that applies only to the corporate and not to the non-corporate entities, and in the healthcare industry, you’ll find largely we have non corporate entities’ (KI 3). In addition, although they are subject to audit, the public sector providers do not face the same accounting regulations. One KI stated ‘Public sector never comes under regulations. No rules or law applies to the public sector. So, there is no level playing field’. Furthermore, the enforcement of cost account submission is weak ‘we are supposed to engage a cost accountant to give us a certificate every year that we have a cost accounting system’ (KI 3), and the cost accounts required are overall line-item accounts that do not address the complex needs of a hospital. Even for the larger facilities, which could face legal action ranging from monetary fine to imprisonment for non-compliance, the KIs were not aware that the government had applied these provisions.

#### Feasibility/willingness for collating and reporting cost information

The interviews also explored the feasibility of setting up a national costing system based on providers cost accounting data as well the willingness of the providers to share their cost information with the government bodies. Three respondents out of 11 were very positive: ‘If the tool is developed, yes, the private sector will be too willing to happily do that’ and that ‘I think by now we are able to get most data from the system’, although one stated a better option would be to ‘ask for volunteers who are willing … You can start with that, that will give you enough sample size’ (KI 6). Conversely, other respondents were of the view that such an effort will not be very useful and moreover will consume resources that could be invested elsewhere ‘if we have to invest in the system that is more detailed and will require more investment, which we would rather, you know, spend on medical equipment, et cetera’ (KI 7).

When asked about willingness to share cost information, it was felt that legislation would be required. Half of the respondents were of the opinion that most private providers would not agree to share or would only share limited data: ‘At the moment, price is available to me, if the cost becomes available to me, then the margin is visible to me …. And it becomes a trade margin-oriented discussion which every provider wants to avoid.’ In contrast, it was agreed that the majority of the hospitals run by NGOs or charitable or philanthropic institutions had no problem with sharing their cost data.

**Figure 3.**
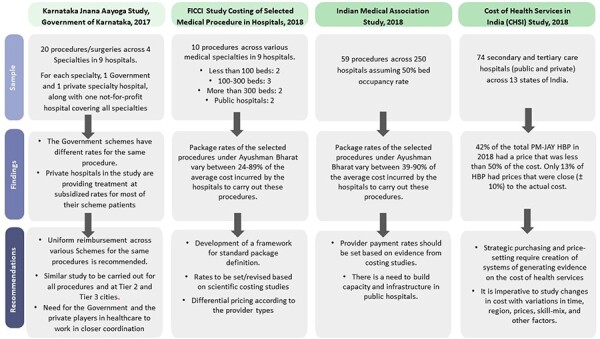
Summary of the previous costing initiative undertaken in India

During our interviews, we identified a felt need for cost accounting to keep a track of costs being incurred, which in turn guides the return on investment and profitability. The most frequently stated and overarching motivation for costing was because ‘I want to know my profits’ (KI 4) and understand the return on investment. Moreover, the providers have started to look into their competitiveness: ‘whether those rates are competitive, whether my efficiency is competitive, whether my procedures are competitive’ (KI 3). A few experts and influential players in the private industry with knowledge of health care costing have been advocating for more use of cost accounting systems to help inform PM-JAY and enable facilities to understand if and which of their costs are being covered, which gave rise to the costing initiatives described earlier and in Figure 3.

On the other hand, we also heard about a lack of interest in cost accounting, since price decisions in private industry ‘are made based on competitive pricing- it all depends on what patients can afford and what [the] market is charging’ (KI 7, 8), ‘so costing is not required’ (KI 8).

#### Challenges to setting up a standardized hospital cost accounting systems for PM-JAY

The interviewees identified numerous internal as well external challenges to establishing cost accounting systems. Amongst the external challenges, one of the issues most frequently (60% of the respondents) highlighted was the stated lack of standardized guidelines or templates available to the hospitals for reporting cost information. Moreover, there are privacy and confidentiality concerns to reporting such data. Amongst the internal barriers were, firstly, the requirement for skilled personnel and a good IT system in the facility. Further, while hospitals have robust health management information systems (HMIS) ‘there’s a challenge, many of the IT systems are not oriented towards the cost’ (KI 1, 4) and ‘how do you integrate it with the financial accounting. So, from billing, how do you segregate? And then paste them into my ledger accounting’. When probed further, we found that even in the facilities where the IT system is good, it is not being used to capture the data relevant to costing, and they tend to have separate financial accounting systems that are not linked to the HMIS: ‘Yeah, so the two key applications we use, one is hospital information system, which is often the billing system, and we have then enterprise application like SAP or, or where we have all our inventories, financial accounting data. So, the combination of both these systems, we do all our financials’ (KI 7). It is likely that building the whole system for small- or medium-sized hospitals will be hard particularly due to the lack of understanding of the usefulness of costing: ‘Doctors don’t understand the impact of costing. When it comes to sharing relevant data, the doctors think that they know, so that’s a problem’ (KI 2). This point was emphasized during the costing panel, where participants underscored that providers generally do not recognize the value of such costing exercises. As a result, the validity of data generated from the existing systems is questionable adding to the reluctance to invest in these time and resource-intensive activities.

#### Using cost information for price setting

While cost-based pricing does not seem to be relevant to the private sector, three respondents mentioned that health care delivery funded by the government sector is likely to be cost oriented, and four respondents expressed that cost-based pricing is more relevant for setting reimbursement rates in publicly financed schemes. ‘… where the government is funding any insurance authorities … they necessarily will look into the cost aspect. Also, they will not approve the rates without looking to the cost of those services and all that … but as for the non-structured, private person has to pay for the cost of their health, for that, why should the hospital apply cost?’ (KI 5).

Five respondents raised concerns over the inadequacy of reimbursement rates for empanelled private provider and recommended that a market survey should supplement the process of price setting.

#### Relationship between government and private health care providers

‘The costing is a very sensitive … data. No corporate entity is willing to share the cost data with [anyone] outside the company … until we have a structured, regulatory framework, the hospital industry is not going to share any type of information easily’ (KI 5). In addition to the issue of sensitivity, interviewees reported that the lack of standardized cost reporting and regulatory frameworks has further accentuated this issue.

A problematic relationship between the private and public sector reason is another reason for unwillingness to share data with the government. The inadequacy of reimbursement rates and the insufficient support provided to the private empanelled providers has further soured the relationship. When asked if they would share their data one KI replied ‘We don’t get any benefit from government. We’ve not got land, you know, we pay 30% duty on medical equipment that we import … we don’t get any subsidy like IT industry gets [like the] tax subsidy for initial five years …. So why should we do it?’ (KI 7); another responded ‘My cost is at least around 12 000. My margin another 3000–4000 Rs., I need at least 15 000 Rs. and you are giving me 8000 Rs. So why do I share my data with you?’ (KI 4). It was also noted that the slow processing of claims data placed a significant burden on the private sector hospitals.

In addition, 4 of the 11 respondents indicated that the private empanelled providers are only included in the rate setting processes in name and the process is not transparent: ‘the Ministry of Health had set up the committee and put 2 or 3 of us across on the committee. They did not …. involve us in anything, and then came up with their own results’ (KI 2).

#### Recommendations for building a national hospital cost system

The respondents made a number of recommendations on how to build efficient cost accounting systems in health care. Firstly, workshops and seminars for both private and public sectors, and including senior staff and management, should be conducted to raise awareness on how cost accounting systems contribute to boosting productivity, efficiency and profit margins.

Secondly, both the IDIs and the panel recognized that government support to developing standardized cost data collection methods and reporting formats, robust information management systems, training on use of cost accounting systems, as well as auditing systems is needed. This support should address in particular the needs of small- and medium-sized health care providers ‘most of the small to medium hospitals sized hospitals cannot invest into the teams which can undertake this detail’ (KI 3). Alternatively, ‘a certain format should be provided to each hospital ….to give the general financial’ (KI 6) and ‘and then at least come up with some kind of a minimum standard requirement’ (KI 8) so that a costing expert can study the costs of small- to mid-sized hospitals. Third, it was suggested that there was a need for more cost accountants to be formally involved in health care cost accounting processes.

Fourth, it was recognized that having a repository of cost data would mean that ‘Everybody would know what is the rate from each hospital. Many hospitals would be willing to participate and they could be the consortium … bringing their own rate’, contributing to more efficient and transparent rate setting processes. Fifth, to improve the trust between the government and private health care providers, government should involve providers in the entire process of reimbursement rate setting and the related data collection and analysis while also implementing trust-building exercises.

Sixth, digitization (2 respondents, KI 3 & 6) is seen as an opportunity to facilitate cost accounting and for the business, if it leads to systems that enable analysis of the profit from different specialities and activities. ‘We now understand that digital is the new frontier … Power BI will help use to see even the cost at the doctors’ level’ (KI 3). The view is that this is not widespread so that hospitals larger than 100 beds ‘Most of them would be electronic’ (KI 6) but others ‘are still, you know, making do with the, uh, basically all handwritten records and accounts’ (KI 6).

Finally, while capacity and trust were seen as an important area to focus on, 80% of the participants also strongly supported that the government should set up regulatory systems or extend existing corporate hospital regulation to all health care providers including both the private and public sectors and small-/medium-sized providers.

## Discussion

Cost accounting can provide health care providers with information to improve efficiency, maximize return on investment and help set tariff rates that reflect an efficient mode of production or service provision. Our qualitative survey to look at the current capacity for cost accounting and cost accounting practices in India found a mixed picture from advanced cost accounting systems in the large, networked hospitals to little documentation of costs among small- to medium-sized hospitals. Among our key informants, there was an acknowledgement of the benefits of cost accounting as well as the limited capacity in the country and awareness of the benefits at the hospital level. They recognized the need to do more in this area to improve health care efficiency and facilitate discussions with the government around setting of evidence-based reimbursement rates that are acceptable to empanelled providers.

In its strategy to achieve UHC, the Indian government has sought the private sector’s involvement through empanelment of private providers (Sarwal *et al*., 2021). There is a huge diversity in the provision of care where around 80% of the care is provided by small- and mid-sized health care providers (up to 400 beds) and the rest by large hospital chains (Federation of Indian Chambers of Commerce and Industry (FICCI), 2018, Assessment of the Healthcare Delivery Market in India, 2023). Our findings indicate that this diversity is manifested by extensive heterogeneity in the existing state of cost accounting, attributable to various provider characteristics including ownership, size, geographical location as well their understanding of cost accounting. While large private providers have their own advanced business management tools and HMIS to inform profitability, HMIS for small- and medium-sized hospitals generally serve a narrower purpose of managing billing and financials. As a result, when collating resource use information from different hospitals, the information is in varied formats and not easily comparable. Existing efforts at compiling standardized costs for public sector planning have involved large-scale costing studies with stand-alone data collection exercises (Figure 3). While providing a good foundation of cost evidence, this approach is not sustainable in the long run. However, government attempts to compile costs at a national level have faced challenges around lack of awareness of the initiative and the availability of the Institute of Cost Accountants standardized template. Only 4 out of 11 participants were aware of the freely available template and guidelines developed by the Institute of Cost Accountants, which all hospitals are required to maintain under the cost audit rules, 2014, but whose use is not enforced by the government.

Where the cost accounting systems in the health care industry in India are rudimentary, this can be explained by lack of costing capacity, the lack of understanding around the benefits of maintaining cost records as well as the use of market rates to help set prices. This in turn means that there is a low perceived benefit of and lack of motivation to introduce cost accounting. The interviews further underscored that the private health care industry remains highly unregulated with weak law enforcement where there is regulation. Along with this, the lack of awareness around costing system standards for health care providers makes the use of cost accounting as standard practice in health care more difficult.

On the other hand, some providers recognize the benefits of cost accounting but the necessary changes to data collection systems pose a potential barrier to implementation. Currently, linking cost information, based on resource consumption, to the HMIS is not a common practice as health care providers do not consider it necessary. Moreover, there is a significant concern regarding the shortage of skilled staff and limited analytical capacity for financial data across the different levels of the health care system. A potential opportunity is to leverage existing and developing digital systems, such as India’s ‘Ayushman Bharat digital Mission’ (ABDM) (NHA, 2024), to establish sustainable cost reporting systems. The ABDM components, including the Ayushman Bharat Health Account for unique individual identification, registration of health facilities and professionals and a unified health interface for interoperability, offer an opportunity to generate critical information for patient-level cost accounting. However, this system will only capture billing information and require additional effort for cost data entry and its integration with the HMIS.

Our findings underline the need to raise awareness around the importance of the information needed for cost accounting and pricing, and strengthening both the capacity of providers as well as the complex relationship between the private sector and government. Efforts should be made to train and recruit skilled staff. One potential step in this direction is to develop a short programme focused on building costing capacity. A programme such as this could be part of an effort to develop a stronger partnership between PM-JAY and its empanelled providers and create a real demand for cost data from both public and private providers. As the private sector already serves 70% of India’s population, it is pivotal for these major stakeholders in health to be aligned (Federation of Indian Chambers of Commerce and Industry (FICCI), 2018). Efforts in this direction could take the form of introducing regulation on costing or creating incentives to produce cost information, e.g., following examples from the UK and Australia where summary cost data for each provider are shared across the sector allowing providers to compare their levels of performance and efficiency.

Government may also be able to leverage consumer interest to promote cost accounting. Currently, consumers lack access to benchmark price information, limiting their ability to make informed choices. Raising public awareness to demand easily accessible, and comparable cost information, at least regarding market prices, could also encourage efficiency and greater transparency for all, and incentivize hospitals to adopt more accountable and efficient practices including cost accounting.

Our findings are consistent with what has been reported by other countries (Guinness *et al*., 2022). There is diversity in how regulatory systems operate in different country settings. Countries such as Australia, England and USA have mandatory cost accounting systems applying to all providers who participate in publicly funded provision, according to the guidelines issued by the public purchaser (Eldenburg and Kallapur, 1997; Tan *et al*., 2011). These are focused at how cost should be calculated for pricing purposes and use standard principles and instructions (InEk, 2007; Department of Health and Ageing, 2011; Serdén and Heurgren, 2011). Establishing such a nationwide cost accounting database also requires consideration of the institutions involved. The Institute of Cost Accountants could play a crucial role in setting professional standards and ensuring the credibility of methodologies for data collection and analysis. However, potential hesitancy from private sector entities to share data with governmental bodies suggests the necessity for an autonomous oversight body that could serve to impartially manage the process, assuaging fears of data misuse and bolstering trust across sectors. Good governance will further demand rigorous validation and auditing protocols to maintain the accuracy and reliability of the data collected.

National standard setting will also face the challenge of the current lack of standardization. There are differences in how different providers collate cost information, which is according to the needs of their set up (Kihuba *et al*., 2016). The availability of a standardized nationwide framework and the establishment of efficient e-HMIS systems to inform the costing will be instrumental in standardization and for cost accounting systems to be used for price setting (Fraser and Blaya, 2010).

There are a number of inherent limitations with the study. Firstly, we followed a snowball sampling approach to identify key informants for our interviews and therefore may have missed some expertise within the field. In addition, the extensive heterogeneity of health care services and providers across India has potential to limit the generalizability of our findings. However, all the respondents were highly experienced national-level stakeholders engaged in health care sector and had knowledge of financial and cost accounting and its role in the hospital sector. Further, they also act as national level representatives for the public and private sectors across rural and urban areas and different health services (hospitals, nursing homes, clinics, diagnostic centres, etc.). The initial analysis was validated by the panel members who expanded the diversity of the group and interviews were conducted until we reached saturation in terms of themes. Secondly, we carried out virtual interviews via Zoom as the respondents were placed in different cities and it was not feasible to travel to each of them. This also meant that our interviews were time bound due to the amount of time available with each respondent, which might have led to missing some aspects.

## Conclusion

In publicly funded health care systems, where resources are finite and public trust is paramount, transparent accounting practices are essential. Cost accounting provides a systematic framework for accurately tracking and allocating costs and generating evidence to inform public sector rates. For the moment, hospital cost accounting in India is rudimentary but highly variable. While there is a recognition of some of the potential benefits, low perceived benefits of adoption and low perceived risks of non-compliance are contributing to the limited motivation to use cost accounting and investment in cost accounting capacity and resources that could inform public sector rates. At the same time, the inadequacy of regulation in this area, concerns about data sharing and non-standardized cost accounting practices are further barriers to the development of a national cost accounting systems.

Establishing a national cost system for India that generates robust credible cost data in a timely and transparent manner will involve working closely with all the stakeholders to develop standardized costing frameworks and templates that take advantage of the digitization of health data, developing the technical capacity of all stakeholders, and an effective regulatory environment overseen by an autonomous institution that is trusted by both payers and providers.

## Supplementary Material

czae040_Supp

## Data Availability

The full data underlying this article will be shared on reasonable request to the corresponding author.

## Reflexivity statement

The authors consist of a diverse team that includes five females and two males and span multiple levels of seniority. Among them, five specialize in health economics, policy and financing in India and two are also experts in global health. Two of the researchers are early career researchers with experience in quantitative and qualitative methods in health economics. In addition, all authors collectively possess extensive experience in health systems research, in lower middle-income countries like India.
