# Supplementary material for: Health care cost accounting in the Indian hospital sector
Source: Health Policy Plan. 2024 May 30;39(7):731–40. doi: 10.1093/heapol/czae040 (PMC11308608; doi:10.1093/heapol/czae040)
Supplement: czae040_Supp [file czae040_supp.zip › suppl_data/Annex 5 Anonymised Panel Summary Report.pdf]

CAN HOSPITAL COST ACCOUNTING CONTRIBUTE TO THE DEVELOPMENT OF A NATIONAL COSTING SYSTEM FOR SETTING PROVIDER PAYMENT RATES FOR PUBLICLY-FINANCED HEALTH INSURANCE SCHEMES IN INDIA

PANEL DISCUSSION

20.06.2023

CONTENTS

EXECUTIVE SUMMARY ____ 2

EVENT AGENDA ____ 4

INAUGRAL SESSION ____ 5

GROUP DISCUSSION: THEME 1 ____ 8

GROUP DISCUSSION: THEME 2 ____ 17

GROUP DISCUSSION: THEME 3 ____ 30

THE WAY FORWARD ____ 34

OUR PANELISTS ____ 36

FURTHER READING MATERIAL ____ 39

EXECUTIVE SUMMARY

The panel discussion, held on 20th June 2023, focused on the role of hospital cost accounting in developing a national costing system for setting provider payment rates under publicly-financed health insurance schemes in India, with a specific emphasis on *Ayushman Bharat Pradhan Mantri Jan Arogya Yojana* (PMJAY). The discussion aimed to understand the importance of cost accounting, identify barriers and facilitators for improving cost accounting systems in the hospital sector, and explore the potential role of the Ayushman Bharat Digital Mission (ABDM) in establishing patient-level cost data collection.

The context of the discussion highlighted the National Health Authority's (NHA) responsibility for implementing PMJAY and regularly updating the list of health benefits packages (HBP) and associated reimbursement rates. The recent use of cost evidence from the study "Costing of Health Services in India" to revise reimbursement rates is a step forward but conducting national-level costing studies is a resource and time intensive activity. This brings forth the need for standardized cost accounting practices but existing hospital-level cost accounting systems in India are not very mature.

The panel discussion addressed three key objectives. Firstly, it sought to emphasize the importance of cost accounting and identify perceived barriers and facilitators for improving cost accounting systems in the hospital sector in India. Secondly, it aimed to identify the best possible methods and processes to establish a sustainable national cost system for setting reimbursement rates under publicly-financed health insurance schemes, particularly PMJAY. Lastly, it explored the potential role of the ABDM in establishing patient-level cost data collection.

The key learnings from the panel discussion emphasized the need for a standardized format and dedicated trained human resources to capture accurate cost information from both public and private providers. Integrating hospital management information systems (MIS) with the ABDM-HMIS system was considered essential for setting up sustainable cost accounting systems and fostering transparency and trust among providers. Sensitizing providers and incentivizing their involvement in sharing hospital information were identified as crucial steps toward building a sustainable cost accounting system.

In conclusion, the discussion shed light on the significance of hospital cost accounting in the development of a national costing system for setting provider payment rates under publicly-financed health insurance schemes like PM-JAY. The discussion highlighted the need for standardized practices, integration of information systems, and provider involvement to ensure transparent, and collection of robust and accurate cost data. These insights will contribute to the establishment of a sustainable and efficient cost reporting system for the Indian healthcare sector.

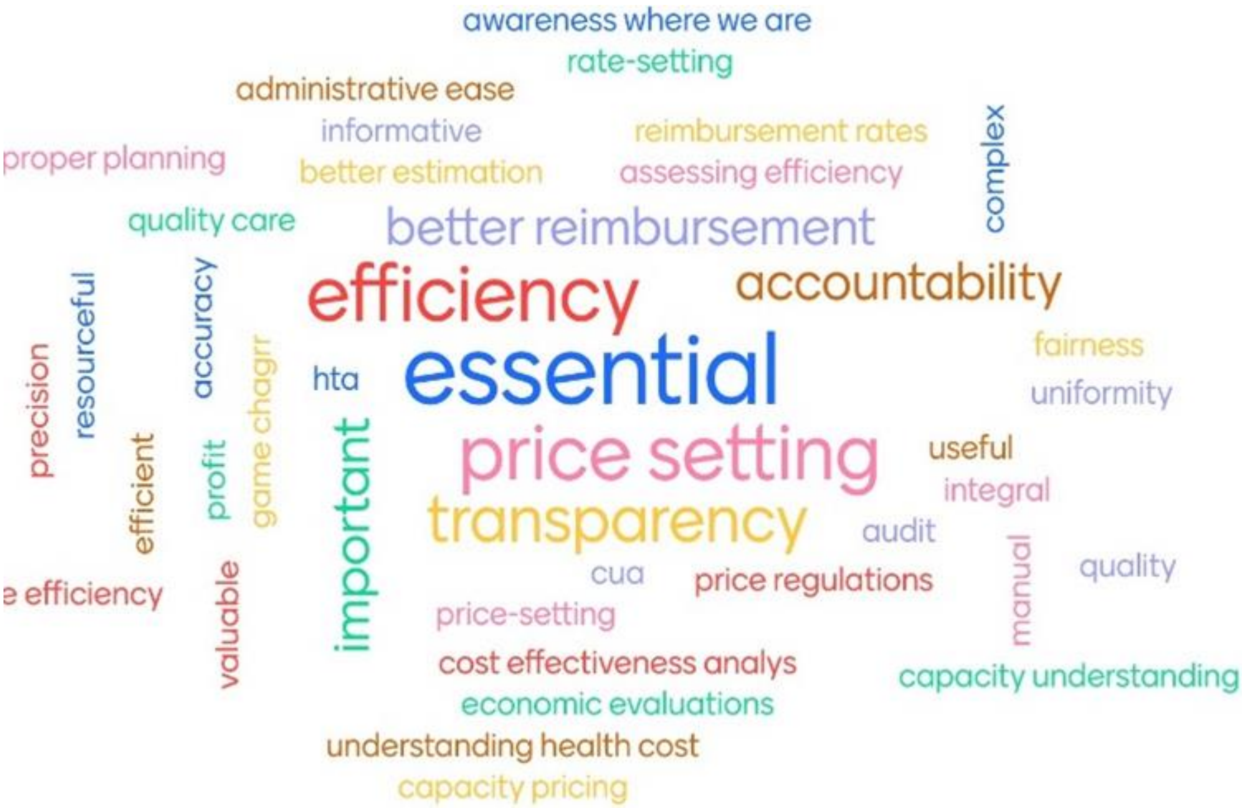

“Empowering India's healthcare system through effective cost accounting: Driving equitable reimbursement rates for accessible, high-quality care."

GROUP DISCUSSION: THEME 1

Session chair

xxxxx

Moderator

xxxxxx

Facilitators

xxxxx

12 Panellists

Discussion themes

- What are the challenges to setting up cost accounting system for hospitals in India?
- What can be done to resolve them?

Cost accounting systems are inevitable and of utmost importance for healthcare sector in India. This necessity arises from the existence of PM-JAY, which is one of the largest health insurance schemes globally, and other state funded health insurance schemes, making the government the largest payer for healthcare services in the country. As the schemes expands, it is envisioned to extend its reach to cover a larger proportion of the population. Nonetheless, this expansion must be achieved within the confines of finite budgets. Hence, it becomes paramount to develop a comprehensive understanding of the costs associated with delivery of healthcare services, which in turn necessitates the establishment of robust cost accounting systems. Nevertheless, the implementation of such systems faces numerous challenges which were highlighted by the panelists and are mentioned below.

Theme 1a: What are the challenges to setting up cost accounting systems for hospitals in India?

Process/health system issues

- The providers do not recognize the need for costing information. This is specifically because the providers have limited awareness on the importance of having access to accurate cost data and potential benefits of cost accounting systems. They do not fully comprehend how cost information can help them in making informed decisions. Furthermore, healthcare professionals primarily focus on delivering patient care rather than delving into the intricacies of cost analysis.

- There is lack of trust and confidence between private providers and the government leading to unwillingness to share data. Private providers perceive sharing cost data with the government as a potential threat to their competitive advantage and an intrusion to their business operations. Costs are a sensitive issue and there are concerns regarding data confidentiality and government interference in pricing decisions. Providers are nervous about the differences in costs which might be due to geographic variations in human resources or capital expenditures.

- There is heterogeneity in size, systems and capacity of different hospitals. Bigger hospitals may need to collect data on a large number of items, whereas comparatively lesser data may be needed in case of smaller hospitals.

- There are differences between public and private sector model of operations.

- There is a lack of investment for establishing sustainable cost accounting systems.

Human resource capacity/training issues

- There is a lack of knowledge, and capacity on costing in general. Individuals do not possess necessary skills and training required to capture and analyze cost data.

- There is lack of awareness around the existing frameworks for costing. The Institute of Cost Accountants of India (ICAI) has developed cost accounting frameworks for various sectors including railways, civil aviation, healthcare, education etc. However, the uptake and application of such systems has been poor by the hospitals.

- Workshops and training on costing and cost accounting systems usually involve higher level executives and decision-makers, while the frontline workers who do the actual work are not involved and adequately trained

- Lack of availability of dedicated HR for cost accounting. There are issues surrounding who would pay for these HR, and whom they will be accountable to.

Costing issues

- Costing is complex, resource intensive and time -consuming activity. It involves intricate calculation, data collection and analysis. The involvement of multiple departments, stakeholders and data sources adds to the complexity, making cost estimation a demanding task.

- Data collection has focussed on clinical side rather than capturing resource utilization and cost information. Largely, the primary focus has been on patient care, and documentation of clinical information on medical or surgical procedures.

- Complexity of cost allocation is a big challenge. Allocating costs accurately and appropriately across various departments, services, and activities required deep understanding of the intricate healthcare ecosystem. Additionally, factors like shared resources, and indirect costs make it challenging to attribute costs accurately to specific services.

- The costs of delivering services varies according to the provider levels and types. Each provider level, such as out-patient clinics, specialty hospitals or tertiary care hospitals offer distinct services and operates with unique resource utilization pattern. Furthermore, different types of healthcare providers, such as public hospitals, private facilities or not-for-profit organizations, have their own cost structures and funding models.

- Costing needs to meet the needs of many purposes – creating efficiency, rate setting (DRG type system), economic evaluations of technologies etc.

- Hospitals generally have a broad understanding of costs, at least at a macro level, but the challenge lies in obtaining accurate cost information at the micro or procedure level. While

hospitals may have systems in place to track overall expenditures, such as facility maintenance, staffing, and equipment, they often face difficulties when it comes to allocating costs to specific procedures or services. This micro-level cost determination involves considering various factors, including direct and indirect costs, resource utilization, and time allocation.

Information systems

- The existence of different IT systems across hospitals poses a challenge to seamless data integration and interoperability. Hospitals usually adopt their own IT infrastructure, including electronic health record (EHR) systems, financial management software, and administrative systems.
- The lack of standardized data management system is primarily attributed to limited investment in cost accounting system. Consequently, data management practices vary across different facilities.
- Hospitals may have a very good record of patients but that is often not linked to the corresponding costs.
- In some hospitals the information of a patient is captured in paper records by medical professionals and the data entry operators may not be skilled with the medical terminologies. Hence information like procedures performed, use of drugs and other consumables may not be recorded in the IT systems. Furthermore, there maybe issues with quality of data and its accuracy.
- In rural hospitals there are internet issues, the implementation of information technology is fragmented along with inconsistent practice of recording the cost and other data. So, paper- based information system is continued.

Theme 1b: What can be done to resolve the challenges

Value proposition

- There is a need to develop shared understanding and the value proposition for undertaking cost accounting. Incentivization can be done in the hospitals for the staff to promote sharing of information on cost.
- PM-JAY can be used as a lever for highlighting the benefits of the costing system.

- Primary design of the system should be for or to serve the purpose of internal improvements/ reporting and efficiency

Process/health system issues

- It is crucial to establish open lines of communication and foster a transparent and collaborative environment between private providers and the government. Building trust requires demonstrating the responsible use of data, assuring data privacy and security measures, and showcasing the benefits and value that can be derived from cost accounting systems. Engaging private providers in the design and development of data-sharing frameworks, ensuring their active participation, and addressing their concerns can help alleviate their reservations and encourage their willingness to share cost data.

- The costing system should not be linked to the payment system (at least initially) as this takes away the focus from setting up robust costing system that informs management of the hospital and promotes efficiency

- Hospitals are less likely to have a problem providing data to a third party. People don't like government surveillance but don't mind others (e.g. government vs google tracking). It is suggested to use a third party to work with providers to do costing and to address issue of costs. Key stakeholders such as Quality council of India (QCI), National Logistic Data Services (NLDS), National Accreditation Board for Hospitals and Healthcare providers (NABH) should be involved. Accreditation of hospitals can be linked to sharing cost information.

- A prudent approach would be to gradually build the process by starting with a few hospitals to showcase the benefits of collecting and reporting cost data. By selecting a small group

of hospitals as pilot sites, their positive experiences and lessons learned can be effectively demonstrated and can motivate other healthcare institutions to join the effort in collecting and reporting cost data. These hospitals can serve as pioneers, highlighting the value and utility of cost data collection and reporting and ensure broader participation in future.

- Given the resource intensive nature of real-time tracking of costs, it is worth exploring whether acquiring cost information from a subset of providers can effectively fulfill the requirements for informing HBPs, obviating the need for comprehensive real-time tracking. This could potentially alleviate the resource strain while still providing valuable insights into the costs necessary for informing HBPs. Nevertheless, striking a balance between the need for timely cost information and the practicality of real-time tracking will be crucial in optimizing the efficiency and effectiveness of cost data collection.

- Lastly, it's important to make a start with a modest framework and work with state governments, private sector agencies that are already working in this domain.

Capacity building/HR

- First and foremost, capacity building programs should be developed and implemented to enhance the knowledge and skills of healthcare providers regarding cost accounting principles and practices. The training programs can be tailored to different stakeholders, including data entry operators, clinicians, administrators, and finance personnel, to ensure a comprehensive understanding of cost-related concepts. Furthermore, awareness should be built to highlight the benefits and relevance of cost accounting in healthcare organizations. Emphasizing how cost data can inform strategic decision-making, resource allocation, and performance improvement can help healthcare providers recognize the value of incorporating cost accounting into their operations.

- There is a need for dedicated HR who possess the necessary expertise to collect, analyze, and manage cost data effectively. To ensure the sustainability of such systems, efforts should be made to retain these skilled individuals through appropriate payment mechanisms and incentives. Creating a supportive and conducive work environment is crucial for attracting and retaining HR personnel, providing ongoing training and

professional development opportunities can further enhance their proficiency and keep them updated with evolving industry practices.

Costing frameworks/templates

- There is a need to build on the institutions and work that is already in place (ICAI templates and operating procedures) rather than starting de novo. The ICAI has designed simple cost accounting tools and processes to the context of the Indian healthcare setting, which can be used.
- There is a need to develop a standardized cost accounting system which has a common framework, standard processes and format that accommodates the diversity of healthcare providers. Furthermore, there is also a need for a standardized approach to cost allocation. By establishing a common approach, healthcare organizations can ensure consistency in cost data collection, analysis, and reporting, enabling meaningful comparisons and benchmarking across providers. This standardization would promote transparency, facilitate resource optimization, and support evidence-based decision-making,

Monitoring/ IT system

- There is a need for a intra-operable platform. A centralized IT platform would promote data standardization, consistency, and quality, ensuring reliable and comparable resource use data.
- Simple IT platform in which you can collect information on all the drugs, consumables and implants (e.g. the drug pilot in Kerala) can be used for public sector. In the private sector, the billing data can be used as a proxy.
- Additionally, efforts should be made to enhance the capacity of healthcare providers in terms of use of infrastructure and technology.

Regulation

- Once the cost accounting system is established then it needs to be linked to the regulatory system in the country.

- There is a need to develop robust data collection and privacy laws that safeguard the confidentiality and integrity of sensitive information. These laws should outline clear guidelines for collecting, storing, and utilizing cost data while ensuring compliance with privacy regulations and protection of provider and patient confidentiality. Additionally, implementing non-disclosure agreements (NDAs) can further reinforce data privacy and confidentiality.

GROUP DISCUSSION: THEME 2

Session chair

xxxx

Moderator

xxxx

Facilitators

xxxxx

10 panellists

Discussion themes

- What are the challenges of reporting full-cost data to a national system?
- What are the mechanisms that could be put in place to encourage provider participation and submission of accurate cost data?

Theme 1: What are the challenges of reporting full-cost data to a national system?

Views on the ongoing hospital cost reporting

- The current hospital cost data reporting activity faces several issues. Firstly, it is a laborious and time-consuming task. Additionally, healthcare providers consider data collection for cost surveillance to be unimportant compared to their primary focus on delivering care.
- Although there is a need for a standardized format in cost surveillance, healthcare providers are reluctant fill out to the information as per the format of the Transaction Management System (TMS).
- Furthermore, the information collected for cost surveillance varies across different healthcare providers.

- Overall, the ongoing hospital cost surveillance activity is hindered by its cumbersome nature, the lack of priority given to data collection by healthcare providers, their hesitance to adopt standardized formats like TMS, and the inconsistency in the information gathered across various providers.

The trust deficit between the providers and the payer

- The presence of a trust deficit between the public and private sectors is a pressing issue that requires attention. It refers to a lack of trust, credibility, and confidence that exists between these two sectors. This trust deficit arises from various factors, including conflicting interests, perceived imbalances in power and resources, and others.
- One of the main challenges in addressing this trust deficit is the reluctance of both sectors to acknowledge and actively work towards resolving it. There may be a tendency to prioritize short-term gains or maintain the status quo rather than invest time and effort into building trust.
- Additionally, systemic issues such as bureaucratic hurdles, regulatory complexities, and differing priorities can further impede progress in bridging the trust gap.

The issue of having a standardized template for cost/price reporting

- Developing a standardized template for cost reporting poses significant challenges due to the diverse characteristics and practices of different hospital providers. The unique nature of each hospital provider, including variations in size, specialization, infrastructure, and patient population, makes it challenging to create a one-size-fits-all template. Hospital providers may have different cost structures, resource allocations, and operational models, which require flexibility in reporting methods.
- Nevertheless, having a standardized template for cost reporting is crucial for several reasons. Firstly, it enables meaningful comparisons and benchmarking across providers, regions, or healthcare systems. Standardized data allows for accurate analysis, identification of cost variations, and the implementation of cost-saving measures. Secondly, a standardized template facilitates transparency and accountability. It ensures that cost reporting practices are consistent, traceable, and auditable, which enhances trust among stakeholders, including policymakers,

regulatory bodies, insurers, and patients. Additionally, a standardized template promotes efficiency in data collection and analysis. By streamlining the reporting process, it reduces the burden on hospital providers and minimizes the resources required for data compilation and verification. This allows healthcare professionals to focus more on delivering quality care rather than spending excessive time on administrative tasks.

The need for capacity building

- The issue of insufficient focus on capacity building becomes apparent when it comes to data entry for hospital cost surveillance. Specifically, there is a lack of emphasis on understanding International Classification of Diseases (ICD) codes, accurate diagnosis coding, and resource use data entry. Effective hospital cost surveillance data entry requires a deep understanding of ICD codes, which are used to classify diagnoses and procedures.
- In addition to diagnosis coding, resource use data entry is another critical aspect that requires focused capacity-building efforts. Properly capturing and documenting the resources utilized during patient care, such as procedures, medications, and services, is essential for calculating accurate reimbursement rates and understanding resource utilization patterns. However, without adequate training on resource use data entry, there is a risk of inconsistent or incomplete documentation, resulting in skewed data and potential financial implications.

Challenges specific to hospital cost data

Collecting hospital cost data faces several challenges that hinder the availability and quality of information. These challenges include:

- *Sensitivity of Private Sector Data:* The private sector often exhibits a reluctance to share sensitive cost data, particularly regarding human resources (HR) salaries and other proprietary information. Privacy concerns and competitive dynamics can limit the willingness of private sector entities to disclose comprehensive cost data.
- *Limited Cost Information in the Public Sector:* The public sector may have limited availability of cost information in the required format. Manual data collection methods

can be time-consuming and error-prone, making it difficult to extract and compile accurate cost data for analysis and decision-making purposes.

- *Lack of Electronic Health Records (EHR) in the Public Sector:* The absence of EHR systems in the public sector poses a significant challenge to cost data collection. EHRs provide a structured and standardized platform for capturing and analysing healthcare-related information. Without EHRs, obtaining disaggregated data, such as drug and consumable costs, becomes challenging.
- *Poor Data Collection Practices:* Inconsistent or inadequate data collection practices contribute to the challenges in obtaining comprehensive cost data. Issues such as missing data or incomplete records can hinder the accuracy and reliability of cost information, making it difficult to derive meaningful insights or perform robust cost analyses.
- *Willingness to Provide Data and Enhance EHR Efficiency:* Encouraging healthcare providers' willingness to share cost data and improve the efficiency of EHR systems is essential. It requires creating a supportive environment where providers understand the value of data sharing for improving healthcare outcomes and cost management. Addressing concerns related to data privacy, data ownership, and the benefits of comprehensive data collection can help foster a willingness to provide accurate and detailed cost information.

Weak legal enforcement around cost accounting

The weak legal enforcement surrounding healthcare cost reporting in India is a pressing concern that demands attention. The current situation raises several issues that need to be addressed to ensure transparency and accountability in the healthcare sector.

- One key area of focus is the implementation and enforcement of the Clinical Establishment Act. While the act aims to register all clinical establishments in India to make it easier to regulate them and implement standard practices, its implementation varies across different states.

- Another issue that needs attention is the auditing of legal frameworks. Presently, the auditing process primarily relies on the turnover of healthcare establishments, which may not provide a comprehensive assessment of cost-reporting practices followed by

different size healthcare organizations. It is essential to broaden the scope of auditing to include small to medium-sized healthcare facilities.

Theme 2: What are the mechanisms that could be put in place to encourage provider participation and submission of accurate cost data?

Making the intent explicit to providers

It is crucial to effectively communicate the importance and intent behind collecting resource-use data to healthcare providers to encourage their cooperation. By understanding the rationale and benefits, providers can actively participate in data collection efforts and contribute to the improvement of reimbursement rates and overall healthcare system efficiency.

- Firstly, providers need to comprehend that the purpose of collecting resource use data is to establish fair reimbursement rates that accurately reflect the costs incurred in delivering patient care. By capturing comprehensive information about the resources utilized, reimbursement systems can be designed to appropriately compensate providers for their services. This understanding helps providers recognize that their co-operation in data collection directly influences the accuracy and fairness of reimbursement rates.
- Furthermore, conveying the significance of resource use data in promoting cost-effective practices is essential. By having access to detailed data on resource utilization, providers can identify opportunities for optimizing their care delivery processes. They can make informed decisions regarding efficient resource allocation, reducing unnecessary expenses, and enhancing the overall quality of care. This understanding empowers providers to actively engage in data collection efforts, as it aligns with their goals of delivering high-quality care while managing costs effectively.
- Another important aspect to emphasize is the transparency and accountability that resource use data brings to reimbursement processes. Providers need to be aware that by collecting this data, reimbursement systems become more transparent and equitable. They can gain insights into how their reimbursement rates are calculated and the impact of their resource utilization on financial outcomes. This fosters trust and

confidence in the reimbursement system, encouraging providers to actively participate and collaborate in data collection initiatives.

- To effectively communicate the intent of resource use data collection, it is essential to engage providers in meaningful discussions, provide clear and concise explanations, and address any concerns or misconceptions they may have. Educating them about the potential benefits, such as fair reimbursement rates, improved cost management, and enhanced transparency, can generate buy-in and foster a sense of shared responsibility in data collection efforts.

Identifying ways to incentivize the providers

Identifying ways to incentivize healthcare providers can play a crucial role in promoting their active participation and cooperation in data-sharing and cost-reporting initiatives.

- *Timely and Faster Reimbursement:* Ensuring that providers submitting cost data have their claims processed within a reasonable timeframe. Receiving reimbursements promptly can be a strong incentive for providers. Timely payments alleviate financial burdens on providers and enhances their cash flow, making them more willing to participate in data-sharing efforts.

- *Incentivization Structure:* Establishing an incentivization structure can be effective in motivating providers to share data and engage in comprehensive cost reporting. This structure can include financial rewards or bonuses based on the quality and completeness of data shared, adherence to standardized reporting formats, and efficient resource utilization. Creating a transparent and fair system that rewards providers for their efforts encourages their active participation.

- *Providing Supportive Structures:* Offering a structured framework and support to providers can help foster their engagement. This can include providing training and resources to enhance their understanding of cost reporting requirements, offering technical assistance in data collection and reporting processes, and creating user-friendly platforms or tools for data entry. By reducing the burden and complexity associated with data sharing, providers are more likely to actively participate.

- *Collaboration and Knowledge Sharing:* Promoting collaboration among healthcare providers, professional associations, and regulatory bodies can incentivize participation in data sharing initiatives. Establishing platforms for knowledge exchange, sharing best practices, and recognizing exemplary performance can create a sense of community and foster a culture of participation.

- *Recognition and Accreditation:* Recognizing providers who consistently demonstrate high-quality data sharing and cost reporting practices can serve as a powerful incentive. Accreditation programs that acknowledge providers' commitment to transparency, accountability, and data sharing can enhance their reputation and create a competitive advantage, encouraging others to follow suit.

- *Feedback and Continuous Improvement:* Establishing channels for provider feedback and actively incorporating their input in the design and improvement of data-sharing processes can enhance their engagement. Providers should have a voice in shaping policies, reporting requirements, and incentives, creating a sense of ownership and accountability.

- *Memorandum of Understanding (MoU):* Implementing a mandatory MoU that requires providers to share data can be considered if incentives alone do not yield the desired results. The MoU can outline the expectations, benefits, and responsibilities of both parties, emphasizing the importance of data sharing for improving reimbursement rates, healthcare management, and overall system efficiency.

Need to be clear on what information is needed

To ensure clarity on the required information for healthcare cost reporting, the following considerations need to be addressed:

- *Differentiating Cost and Price Data:* It is important to clarify whether cost data or price data is needed for rate-setting purposes. Cost data refers to the actual expenses incurred by hospitals in delivering healthcare services, including direct costs (e.g., materials, personnel) and indirect costs (e.g., overhead expenses). Price data, on the other hand, pertains to the charges or prices assigned to specific services. It is crucial to identify which type of data is provided to inform the rate-setting process.

- *Methodology for Costing:* A standardized and well-defined methodology for costing needs to be identified. This involves establishing clear guidelines and protocols for cost allocation, identifying cost drivers, and determining appropriate methods for calculating costs. Having a standard methodology ensures consistency and comparability in cost reporting across different healthcare providers.

- *Analyzing the Role of Capacity, Volume, and Geography:* It is essential to analyse the impact of capacity, volume, geography, and location on cost reporting. Different healthcare facilities may have varying capacities to provide services, which can influence their cost structures. Additionally, the volume of services delivered and the geographic location can contribute to cost variations. Analyzing these factors helps in understanding the nuances of cost data and ensures accurate comparisons.

- *Challenges in Collecting human resource Allocation Data:* The collection of data pertaining to HR allocation can be challenging but is crucial for accurate cost reporting. HR expenses constitute a significant portion of healthcare costs, and understanding how resources are allocated across departments and services is essential. Efforts should be made to develop standardized methodologies for capturing and reporting HR allocation data to ensure its accuracy and reliability.

- *Submission of Billing Data:* It is pivotal to understand that there are concerns regarding the sensitive cost data and the need of expertise required for cost data collection. In the absence of that, or until cost surveillance systems develop, the hospitals should be required to submit comprehensive billing data. This includes details of services provided, procedures performed, medications administered, and other relevant billing information. Collecting billing data alongside cost data provides a comprehensive view of resource utilization and aids in accurate cost estimation.

Developing a robust and flexible template for cost reporting

- To address the challenges associated with implementing a standardized template, it is important to adopt a flexible approach. The template should allow for customization or additional modules that capture the unique characteristics of different providers.

Collaboration between stakeholders, including hospitals, policymakers, and industry experts, can help develop a template that strikes a balance between standardization and flexibility.

- Regular review and updating of the standardized template are also essential to accommodate evolving healthcare practices, new cost components, and emerging trends in the industry. This ensures that the template remains relevant, accurate, and aligned with the changing landscape of healthcare delivery.

Addressing the gaps in capacity building

- Addressing capacity-building gaps is crucial to ensure the integrity and usefulness of hospital cost surveillance data. Focussed training programs should be designed to enhance the understanding of ICD codes, diagnosis coding practices, and resource-use data entry techniques.
- Regular assessment and feedback mechanisms should also be implemented to monitor the effectiveness of capacity-building initiatives and identify areas that require further improvement. This iterative approach ensures that individuals responsible for DRG data entry are continuously supported and equipped with the necessary skills to perform their tasks effectively.
- Furthermore, capacity-building efforts should not be seen as a one-time activity. Continuous training and upskilling opportunities should be provided to keep individuals updated with the evolving methodologies and best practices in cost data management. This can be achieved through workshops, seminars, online resources, and knowledge-sharing platforms.

Strengthening legal enforcement

- It is crucial to strengthen the legal enforcement mechanisms related to healthcare cost reporting. This can be achieved through the imposition of stricter penalties for non-compliance, the establishment of specialized regulatory bodies responsible for monitoring and enforcing cost reporting requirements, and conducting regular audits and inspections to detect any discrepancies or violations.

- Additionally, raising awareness and providing education on the importance of accurate cost reporting and the legal implications of non-compliance is essential. Through awareness campaigns and educational programs, we can foster a culture of compliance and encourage healthcare providers to take responsibility for accurate cost reporting.

- Collaboration with professional associations representing healthcare providers is also crucial. By engaging these associations, we can develop guidelines, provide training programs, and foster a collective commitment to compliance with cost-reporting requirements. Working together with these associations can promote a culture of transparency and support the implementation and enforcement of effective legal frameworks.

The way forward to setting up sustainable hospital cost surveillance

- *Identifying an Ideal Sample Frame for hospital cost surveillance:* For identifying providers who can participate, it is important to have an ideal sample frame that captures key variations in healthcare settings. While a large sample may not be necessary, a good sample with quality data should measure variations such as labor costs, teaching status, and urbanicity. Encouraging market consolidation and driving efficiency should be the focus, possibly with phased implementation to address political considerations.
- *Identifying the factors Outside the Control of Providers:* It is important to identify the factors that are not in the control of the hospital managers. Labour costs are typically not directly within the control of managers due to external factors like labour regulations and market conditions, it is still important to identify variations in labour costs as they significantly contribute to overall healthcare expenses. Understanding these variations can help healthcare systems identify areas where interventions or policy changes may be needed to address cost disparities and promote efficiency. By distinguishing between factors that are within the control of hospital managers, such as equipment and drug costs, and those that are outside their control, like labour costs, healthcare systems can develop targeted strategies to optimize cost management. This approach recognizes the influence of different factors on healthcare costs and empowers managers to make informed decisions to reduce expenses within their purview while addressing broader systemic challenges.

- ***Start with Simple/Basic Cost Data:*** Starting with a simple and basic cost data reporting system is a proven approach used by many countries. This approach helps in attracting a larger client base and allows for continuous improvement based on feedback from providers. Expecting multiple versions of the template in the initial years is normal, as it helps refine the reporting system. Over time, this iterative process leads to the development of a national cadre of experts who have grown alongside the system.
- ***Audit:*** Conducting audits on a small sample of participating hospitals helps ensure accuracy and compliance with cost reporting. Auditing can provide valuable insights and feedback for improving the reporting process.
- ***Supplementary Use of Charge Data:*** While charge data (billing data) can be used to supplement gaps in information, it is important to be cautious. Charges tend to be biased in favor of large, urban facilities.
- ***Provider Participation:*** There are various approaches to encourage provider participation. Mandating participation through contracts, providing financial incentives, or providing performance-related information back to the provider can be effective strategies. In Australia, participation is voluntary for private providers, and the Independent Pricing organization provides aggregated feedback on cost management with benchmarks to all providers without disclosing individual names or identifiers. Such feedback can highlight good performance, influence future behavior and drive improvements.
- ***Transparent Drafting and Public Review:*** When developing costing numbers and prices, it is crucial to promote transparency. Making the draft available publicly, along with explicit formulas, and allowing a certain time period to comment encourage stakeholder engagement. This approach builds trust, fosters transparency, and often generates valuable input and intelligence.
- ***Impact Analysis and Phased Implementation:*** Before implementing new templates or cost measures, it is advisable to conduct an interim analysis to understand the performance of the providers in reporting the cost data. Further, a phased implementation process can be considered to manage the transition effectively.
- ***Establish a Standing Round Table of Experts:*** Establishing a standing round table of cost experts can provide ongoing guidance and insights. Involving experts from

countries like Thailand, Australia, United Kingdom and others with established costing systems would be valuable.

- ***Development of Medical Pricing Index:*** The development of a Medical Pricing Index can be beneficial. A Medical Pricing Index serves as a benchmark for determining the appropriate pricing of medical services and treatments. It takes into account various factors, including input costs, technology advancements, and market dynamics, to establish fair and reasonable pricing standards. Implementing a standardized and transparent Medical Pricing Index helps ensure consistency, fairness, and affordability in healthcare pricing, benefiting both providers and patients.
- ***Accounting for Return on Investment:*** It is crucial to account for return on investment (ROI) in the healthcare sector, particularly for the private sector. Including ROI considerations in rate-setting enables private healthcare providers to factor in their investments and operational costs when determining the overall costs of healthcare services. This approach helps create a more accurate and comprehensive understanding of the financial aspects involved and encourages private sector participation in cost reporting initiatives.

GROUP DISCUSSION: THEME 3

Session chair

xxxxx

Moderator

xxxx

Facilitators

xxxx

10 panellists

Discussion themes

- Is there potential to use ABDM-compliant hospital management information systems (HMIS) for PMJAY cost reporting?
- How could this be done?

Theme 1: Is there potential to use ABDM-compliant hospital management information systems (HMIS) for PMJAY cost reporting?

Challenges/ Concerns:

1. Digitized hospitals have been collecting essential data similar to what the ABDM-HMIS system gathers, including length of stay. However, the challenge arises when hospitals are required to provide this information once again in a separate system. This duplication of data submission places an additional burden on hospitals within the cost surveillance system. Since all the necessary attributes are already captured, there

should be an effort to eliminate the need for redundant data submission and alleviate the burden on hospitals.

2. There are two groups in the healthcare industry: the larger hospitals that calculate and determine costs based on various parameters, and the majority (60-70%) comprising of smaller and medium-sized hospitals that are more service-oriented. The latter group lacks a proper costing methodology, resulting in limited knowledge of their earnings and expenditures.
3. Smaller hospitals lack awareness of costing practices, highlighting the importance of involving representative organizations such as the Hospital Board of India for training. By providing adequate training, healthcare providers can contribute high-quality data.
4. Prices for medical procedures vary based on the type of insurance coverage, such as public insurance like PMJAY, private insurance, or no insurance. This difference in insurance coverage leads to variations in billing for each procedure.
5. There is a need to develop a common detailed standardized template for uniform data collection from all the hospitals.
6. The uptake and diffusion of ABDM are crucial, particularly as its adoption in private facilities is currently progressing slowly.
7. It is essential to establish different price rates for public and private healthcare facilities, and ensure transparency in the process. However, there is a significant disparity in the understanding of cost and price among different types of healthcare providers.
8. Costing of consumables like drugs depends on the procurement e.g., if pharmacy is outsourced or in-house. There is a need for the laws for re-usable consumables and the costing process should be modified accordingly.
9. Private hospitals have distinct systems in place, including billing systems, Electronic Medical Records (EMR), and material management. To capture real-time data effectively, integration of these three systems is necessary. Currently, cost is being captured in the hospital's MIS. However, there is a lack of standardized format for entering data in the HMIS.
10. A more effective approach would be to focus on costing individual treatments or procedures rather than entire packages. Capturing granular information about costs can be challenging.

Theme- 2: How could this be done?

Benefits/ Suggestions:

1. ABDM aims to implement its system and offer digital health solutions to the majority (60-70%) of providers lacking HMIS, thereby enhancing effectiveness. Additionally, capturing data at the hospital level beyond length of stay and diagnosis holds great interest.
2. We will get real time granular data from all the hospitals. However, there is a critical need for a standardized format to enable seamless aggregation and integration of data at the national level.
3. Cost surveillance is currently of great importance. When planning for cost surveillance initiatives, valuable lessons from other countries like Taiwan and Singapore, where providers were actively involved, should be considered. For instance, in Taiwan, the government allowed providers to participate in developing the template for cost surveillance. Also, financial incentives have also helped in terms of the uptake of these systems in some other countries.
4. The need for qualified and trained personnel in the field is crucial to ensure proper data collection. Additionally, with the implementation of ABDM, there is a push towards an automated system that heavily relies on the digital architecture that has been established.
5. Clarity is essential for the private sector to avoid unnecessary allocation of manpower and resources in figuring out operational aspects. Currently, only consultants are being consulted for determining the base rate, but involving hospital management in this process is crucial for comprehensive decision-making.

Overall theme and summary remarks:

Is ABDM-compliant hospital management information systems (HMIS) and Health claims exchange specification (HCX) are a way forward in setting up a sustainable cost surveillance system?

a. *Comparison with the current system:* The group unanimously agreed that the new system offers a substantial improvement over the current one. It stands out by providing detailed patient-level information instead of just overall cost estimates. The data entry

process is automated, eliminating the need for repetitive manual input. In addition to data on health-related resource use, the collected data could potentially generate other valuable insights from the hospital's overall operations which could prove to be beneficial for the institution.

b. *Adequacy of the current system:* The current hospital system, which comprises EMR systems, HIS systems, and material management systems, is considered adequate for digital public good. However, the ABDM complaint HMIS system stands out due to its extensive and specific information regarding the recurrent resources utilized in patient care. This information can be utilized for costing purposes. Costing requires data on both resource quantity and price. The MIS systems can reliably provide information on quantity for most parameters, while price information is slightly more sensitive and may require careful handling.

c. *Heterogeneity in providers:* There is a significant variation in the nature of hospitals, which affects the availability and quality of cost accounting systems. Factors such as hospital type and bed capacity play a role in determining the sophistication of these systems. Large hospitals generally have robust cost accounting systems, while small and medium-sized hospitals often lack such systems and may struggle to provide detailed cost information.

d. *Standardized source and template:* The group reached a consensus that there is a requirement for a standardized source or framework to obtain accurate information on the price of input resources and their costing. Tracking variations in prices can be incorporated into cost analysis. This standardization can also help build trust between healthcare providers and payers. Additionally, there may be a need for mentoring and support to assist hospitals in effectively providing this information.

e. *Incentivization:* In order to encourage healthcare providers, who are the end users responsible for providing the information for costing and price setting, to adopt the new system, a value proposition needs to be established. This means that incentives should be offered to hospitals, as they are the ones providing the information. While specific incentives were mentioned, there is also potential for additional incentives that go beyond the current proposals.

THE WAY FORWARD

To establish sustainable healthcare cost accounting systems in India, several steps need to be taken. First, there is a pressing need for a standardized format that enables the collection of cost information. In addition to standardization, creating transparency, trust, and partnerships among various stakeholders is crucial. This can also be achieved by promoting the adoption of the standardized format and encouraging collaboration between public, private, and regulatory authorities. By doing so, a unified approach can be developed towards cost surveillance, ensuring better coordination and understanding among all parties involved.

Addressing the mindset of healthcare providers is another critical aspect. Efforts should be made to sensitize them to the benefits of evidence-based price setting. This can be accomplished through training sessions and the development of Information, Education, and Communication (IEC) materials. Furthermore, it is crucial to encourage private healthcare providers to actively participate in the process of costing, both for their own benefit and for the overall national costing system. By demonstrating the value to their own businesses and offering appropriate incentives, their participation can be encouraged, leading to a more comprehensive and accurate representation of healthcare costs.

To streamline the process of retrieving costing data, the adoption of automated systems is recommended. Digitization can play a vital role in facilitating the efficient collection and analysis of cost information. Insurance companies can particularly benefit from this approach, as it enables them to create selective insurance slabs based on the severity of diseases. By leveraging digital solutions, the insurance system can be tailored to meet the specific needs and costs associated with different health conditions.

In conclusion, the way forward for establishing sustainable healthcare cost accounting systems in India involves implementing a standardized format for data collection, integrating it with the existing HMIS, fostering transparency and partnerships, getting the healthcare providers on board and incentivizing them, adopting automated systems for data retrieval, and synchronizing reimbursement rates with the Health Benefit Packages. By following this comprehensive approach, India can develop a robust and sustainable system for monitoring healthcare costs effectively.

Potential actionable steps:

1. A consultation process involving providers and payers to develop and recommend costing principles and templates that effectively address their needs.
2. Form a working group comprising a diverse range of stakeholders, including private and public providers, insurers, academia, ABDM, and NHA/SHAs for setting up sustainable costing system and drive its implementation.
3. Develop a comprehensive capacity building program for providers that highlights the value proposition of costing aimed to enhance their understanding of the importance of accurate costing and provide them with the necessary skills and knowledge to participate effectively.
4. Initiate a pilot or demonstration costing program that utilizes the agreed templates and the ABDM HMIS as a practical implementation of the costing system, allowing stakeholders to test and refine the templates and processes.

FURTHER READING MATERIAL

1. Prinja S, Brar S, Singh MP, Rajsekhar K, Sachin O, Naik J, et al. Process evaluation of health system costing-Experience from CHSI study in India. PLoS One. 2020;15(5):e0232873.
2. Prinja S, Singh MP, Guinness L, Rajsekar K, Bhargava B. Establishing reference costs for the health benefit packages under universal health coverage in India: cost of health services in India (CHSI) protocol. BMJ open. 2020;10(7):e035170.
3. Singh MP, Popli R, Brar S, Rajsekar K, Sachin O, Naik J, et al. CHSI costing study-Challenges and solutions for cost data collection in private hospitals in India. PloS One. 2022;17(12):e0276399.
4. Singh MP, Prinja S, Rajsekar K, Gedam P, Aggarwal V, Sachin O, et al. Cost of Surgical Care at Public Sector District Hospitals in India: Implications for Universal Health Coverage and Publicly Financed Health Insurance Schemes. PharmacoEconomics-Open. 2022;6(5):745-56.
5. Chauhan AS, Guinness L, Bahuguna P, Singh MP, Aggarwal V, Rajsekhar K, et al. Cost of hospital services in India: a multi-site study to inform provider payment rates and Health Technology Assessment. BMC health services research. 2022;22(1):1-12.
6. HTAIn Regional Resource Centre Department of Community Medicine and School of Public Health. A Handbook of Health System Costing. Chandigarh, India; 2021.
7. The National Health System Cost Database for India (NHSCD) provides a platform for cost information for healthcare decision making in India. It can be accessed at: National Health System Cost Database for India, Department of Community Medicine [cited Jun 26, 2023]. Available from: https://pgicostdatabase.co.in/.
8. The National Health Authority (NHA) released consultation papers on the Provider Payments and Price Setting; Value- based to value Based Care: Ensuring better Health Outcomes and Quality Healthcare under Ayushman Bharat Pradhan Mantri Jan Arogya Yojana (AB PM-JAY). It can be accessed at: National Health Authority [cited Jun 26,2023]. Available from:https://pmjay.gov.in/sites/default/files/2022-03/AB%20PMJAY%20Price%20Consultation%20Paper_25.03.2022.pdf;

https://abdm.gov.in:8081/uploads/VBHC_Policy_Document_For_Upload_a20f871a55.pdf.

9. Yashika Chugh, Lorna Guinness, Abha Mehndiratta, Shankar Prinja, and Javier Guzman "The Value of Investing in Cost Data–Lessons from Health Systems Costing Repository in India" Available at : https://www.cgdev.org/blog/value-investing-cost-data-lessons-health-systems-costing-repository-india.
10. Lorna Guinness , Srobana Ghosh , Grace Achungura , Javier Guzman and Abha Mehndiratta "Generating and Using Cost Evidence to Inform Provider Payment Rates: Lessons from High-Income Countries for India's National Health Insurance Program" https://www.cgdev.org/publication/generating-and-using-cost-evidence-inform-provider-payment-rates-lessons-high-income.
11. Lorna Guinness , Srobana Ghosh , Javier Guzman and Abha Mehndiratta "How Does a Hospital Costing System Support Progress Towards Universal Health Coverage?" https://www.cgdev.org/blog/how-does-hospital-costing-system-support-progress-towards-universal-health-coverage.
12. Sarah L Barber, Luca Lorenzoni and Paul Ong "Price setting and price regulation in health care. Lessons for advancing Universal Health Coverage". Available at :https://www.oecd.org/health/health-systems/OECD-WHO-Price-Setting-Summary-Report.pdf.
13. Akashdeep Chauhan Singh , Lorna Guinness , Srobana Ghosh , Deepshikha Sharma , Abha Mehndiratta , Javier Guzman and Shankar Prinja "Building a Better Evidence Base for Health Technology Assessment: Lessons From India". Available at : https://www.cgdev.org/blog/building-better-evidence-base-health-technology-assessment-lessons-india.

****
