# Supplementary material for: Health care cost accounting in the Indian hospital sector
Source: Health Policy Plan. 2024 May 30;39(7):731–40. doi: 10.1093/heapol/czae040 (PMC11308608; doi:10.1093/heapol/czae040)
Supplement: czae040_Supp [file czae040_supp.zip › suppl_data/Annexure 1.docx]

**Healthcare cost accounting in the Indian hospital sector**

**Annexure 1: Definitions**

| **S. No.** | **Terms** | **Definitions** |
| --- | --- | --- |
| 1. | Activity-Based Costing (ABC)^1, 2^ | Activity-based costing (ABC) is a methodology for more precisely allocating overhead costs by assigning them to activities. The basis for ABC is a belief that all activities exist to support the production and delivery of goods and services and that all indirect costs can be traced and allocated to individual products and services. It is widely adopted in public and private, service and managerial organizations and provides more accurate picture of ‘true cost’. |
| 2. | Activity-Based Management (ABM)^2^ | Activity-based management (ABM) can be defined as the entire set of actions that can be taken on a better-informed basis using ABC information. The aim is to achieve the same level of output with lower costs. |
| 3. | Time-driven ABC^2^ | Time-driven activity-based costing is an attempt to overcome some of the weaknesses associated with ABC. TDABC differs from traditional ABC, in that time is used as the primary cost driver. The assumption underlying the TDABC method is that most resources (i.e. manpower, equipment, and facilities) have capacities that can be measured in terms of time. It is easier to implement than ABC |
| 4. | Target Costing^3, 4^ | Target costing is a concept that takes the prices paid for healthcare services as a given and then determines the cost structure necessary for financial survival given the prices set. |
| 5. | Balanced Scorecards^5, 6^ | It is a strategic performance management tool that emphasized measurement from multiple perspectives. BSC is driven by, and aligned with, the organization’s mission, vision, and strategy by selecting measures or key performance indicators (KPI), that fulfil few requirements. |

**References:**

1. <https://www.accountingtools.com/articles/activity-based-costing>.

2. Carroll N, Lord JC. The Growing Importance of Cost Accounting for Hospitals. J Health Care Finance. 2016;43(2):172–85.

3. <https://quizlet.com/299950591/chapter-5-healthcare-finance-key-concepts-flash-cards/>.

4. Raulinajtys-Grzybek M. Cost accounting models used for price-setting of health services: An international review. Health Policy. 2014 Dec 1;118(3):341–53. .

5. <https://balancedscorecard.org/bsc-basics-overview/#:~:text=The%20Balanced%20Scorecard%20was%20originally,as%20the%20measure%20of%20success>.

6. Ax C, Bjørnenak T. Bundling and diffusion of management accounting innovations—the case of the balanced scorecard in Sweden. Manag Account Res. 2005 Mar 1;16(1):1–20.
