# Supplementary material for: Health care cost accounting in the Indian hospital sector
Source: Health Policy Plan. 2024 May 30;39(7):731–40. doi: 10.1093/heapol/czae040 (PMC11308608; doi:10.1093/heapol/czae040)
Supplement: czae040_Supp [file czae040_supp.zip › suppl_data/Annexure 2.docx]

**Healthcare cost accounting in the Indian hospital sector**

**Annexure 2: List of Organisations of the participants of the virtual panel**

1. Ministry of Finance, Government of India
2. Department of Health Services, Kerala, India
3. World Health Organisation
4. Department of Health Research, Ministry of Health and Family Welfare, India
5. National Health System Resource Centre, New Delhi, India
6. London School of Hygiene and Tropical Medicine, London, United Kingdom
7. Health Economics and Health Technology Assessment (HEHTA), University of Glasgow, United Kindom
8. LV Prasad Eye Institute, India
9. Health System Transformation Platform
10. Center for Global Development, Europe
11. Association of Healthcare Providers India
12. Sree Chitra Tirunal Institute for Medical Sciences and Technology (SCTIMST)
13. National Health Authority, New Delhi, India
14. Ayushman Bharat Digital Mission, National Health Authority, New Delhi, India
15. Kalam Institute of Health Technology, India
16. State Health Agency, Haryana, India
17. Fortis Healthcare, India
18. Jan Swasthya Sahyog, India
19. Indian Medical Association
20. Jawaharlal Institute of Postgraduate Medical Education and Research, India
21. King’s College London
22. National Institute of Epidemiology, India
23. The World Bank
24. ACCESS Health International
25. Indian School of Business, India
26. State Nodal Agency, Chhattisgarh, India
27. Aditya Birla Health Insurance Company Limited
28. Confederation Of Indian Industry
