# Supplementary material for: Health care cost accounting in the Indian hospital sector
Source: Health Policy Plan. 2024 May 30;39(7):731–40. doi: 10.1093/heapol/czae040 (PMC11308608; doi:10.1093/heapol/czae040)
Supplement: czae040_Supp [file czae040_supp.zip › suppl_data/Annexure 3.docx]

# **Healthcare cost accounting in the Indian hospital sector**

# **Annexure 3: Interview tool guide**

#### Background

The National Health Authority (NHA) is responsible for implementing India’s flagship public health insurance scheme [Ayushman Bharat Pradhan Mantri Jan Arogya Yojana](https://en.wikipedia.org/wiki/Ayushman_Bharat_Yojana) (AB-PMJAY) at the national level. One of the major responsibilities of the NHA is to regularly update the list of health benefits packages (HBP) - predefined packages of care for different conditions included under the AB-PMJAY and to set appropriate prices for these HBPs. Based on the prices or provider payment rates set by NHA, empaneled hospitals are reimbursed. The HBPs have evolved through a process of review of pre-existing insurance programs and consultation with key stakeholders. This system is now looking to reform due to some of its challenges (eg. adequacy of reimbursement rates, no adjustment of case-mix).

Diagnosis-related group (DRG) based payment mechanisms have been adopted by several countries to create an effective framework for monitoring the quality of care and the utilization of services in a hospital setting. To address challenges in the PMJAY payment scheme and enhance efficiency and sustainability, India is currently trialing the transition from case-based to DRG-based payment systems. The setting of DRG reimbursement rates requires information on both a base rate for health services provision and DRG (severity) weights.

Cost accounting systems are pivotal tools to determine the cost of each service or product used in patient healthcare. They explain the production processes, how this links to costs, and provide detailed information to be used for analytics and decision-making which is one way to help understand the level of efficiency. Looking to the future of PMJAY, it is imperative to understand and therefore standardize cost-accounting practices so as to define methods to estimate and revise both the base rate and price weights for DRGs. The following discussion aims to get insights into existing cost accounting practices in the hospital sector in India to develop an understanding of the role of cost accounting in public and private health sectors to help inform strategies for improving the cost evidence base for the setting of reimbursement rates. We also seek information on the importance, perceived barriers, and facilitators to standardize cost accounting systems in the health sector in India and how this might vary across different types of providers/ provider networks, between the public and private sectors, and in different settings.

#### Objectives of the key informant interviews

To understand cost-accounting practices and their role in hospitals (public/private/empaneled) delivering healthcare in India in different settings in India.

| ***The interviewer to provide the consent form and request the participant to sign this and agree to the interview being recorded.*** |
| --- |

#### Part I: Participant Information

1. Name

2. Organization

3. Designation

4. Roles and Responsibilities

5. Roles and responsibilities in relation to the DRG pilot

#### Part II: Interview tool

| **Interviewer to prompt where appropriate for differences between different types of facilities** (public/private/empaneled/large/small) and different settings (state, remote vs urban) |
| --- |

1. We want to ensure that we both understand the terminology in the same way. What do you understand by the term “cost accounting”?

| **Interviewer to introduce the Working Definition of cost accounting for the purposes of the interview/study:**  Cost accounting is the process of estimating and classifying costs incurred by an organization. In healthcare organizations, cost accounting is used to determine the cost of each service or product used in patient care, providing detailed information that can be used for analytics and decision-making. Combined with other charges on a patient’s transaction record, a complete picture of a patient visit emerges. The data can be compared to payer rates to determine whether costs are in line with expected reimbursement to make pricing decisions. The same data can be parsed a different way, aggregated by the provider, for example, to assess the provider’s performance against peers. Cost data is also useful in facilitating staffing decisions, informing capital request decisions, exploring new services, and more. |
| --- |

1. Are you familiar with any specific cost accounting systems in the hospital sector in India? Please can you describe the system that you are most familiar with.

| **Hints**: What data is collected; how is the information organized; what resources (HR/otherwise) are required to manage and implement the system. |
| --- |

1. What purpose do the management accounting/cost accounting systems serve in the hospital sector in India (e.g., reporting only; management tool; price/charge setting?)

| **Hints**   - Is it just to comply with Cost accounting record and audit rules 2014 ([CARR](https://icmai.in/upload/Students/Circulars/Companies-Rules-2014.pdf))? - To submit product-wise cost sheets to the Ministry of Company Affairs (MCA). - For Internal management such as calculation of cost/profit, center-wise conversion cost based on direct cost and total cost approach - To prepare product/activity-wise cost statement - To improve efficiency/ To draw inferences on cost comparisons across locations, trends, and relative profitability. - Effective resource management (resources cost minimization, optimization of resource use levels) - Management processes (e.g. Improve decision-making process in hospitals) - Setting of prices/rates/charges/billing (Calculation of patient-wise cost) - Operating performance (measuring results relative to the assets used to achieve those results) - Estimating the capacity of each resource? Changing the allocation of different resources? - Modify, or discontinue a service or program? - Making cost projections/ budgeting? - Others? |
| --- |

1. Are there any regulatory requirements in respect of reporting cost accounts for the health sector that you are aware of? If yes, what are the current regulatory requirements? Which authority is responsible for the regulation? And does this vary across different types of provider organizations/settings?

| **Hints**   - How are costs generally designed and identified in the Indian healthcare industry? How are these costs grouped? (Costs related to main medical departments, medical support department, non-medical department, direct/indirect/overhead allocation) - can you refer us to any templates/guidelines? - Can you tell us about the variation in cost reporting requirements for different hospitals in India? If yes, do you know if these are in any way related to the yearly turnover of the hospitals? Can you explain your answer in detail? - Are the requirements the same for empaneled and non-empaneled hospitals? - Does cost accounting requirement vary for private and public hospitals? - Is there any standardized template for healthcare organizations? If yes, then can you please elaborate or provide headings, if this template is different for empaneled, private, or not-for-profit health providers? - If yes, can you tell us broadly about the minimum standards that provider organizations adhere to? - The minimum standards used by healthcare organizations in context with the Indian health system, how is it different from the standards followed by other countries? |
| --- |

1. Can you name a few challenges concerning administering these requirements and collecting information on costs? How do these vary across different types of provider organizations? (Applicable if the answer to Qs 4 is Yes)

Hints: Data systems, human resources capacity, IT Support, Lack of regulatory/legal guidelines

1. What are the consequences of non-compliance and can you tell us about any national authority or agency that is responsible for this? (Applicable if the answer to Qs 4 is Yes)

Hints: Who checks? How regularly? What are the fines/accreditation impacts? Reputational risk?

1. What are common cost reporting systems (internal management tools) used by provider organizations/hospitals in India and how do they vary across different types of provider organizations?
2. What are some of the internal software or systems used by healthcare provider organizations?
3. Is there a platform or software available for the hospitals with standardized templates for cost accounting integrated with the costing method? If yes, is it available online, or could you please explain the different headings under which the cost data is being reported?
4. There are some guidelines and formats provided by MOH relating to the submission of cost information for all hospitals. Are you aware of these documents? How are these guidelines beneficial in the healthcare industry? Are these templates being used? If yes, then how? If No, then why?
5. What are some of the factors that influence the cost of data collection and reporting in different healthcare organizations? Does the size of the facility, number of procedures or availability, or level of treatment influence the cost reporting system?
6. How do you think that cost accounting practices vary in different hospital provider organizations? What drives these differences?

- Do the practices vary among public, private, or empaneled provider organizations? Rural/urban? Remote?
- Is the type of accounting method used affected by the size of the health facility and the extent of utilization of the services that are being provided? Further, is it that the cost accounting process will vary in the for-profit and not-for-profit healthcare organizations?
- If so, what could be the underlying factors causing this difference in choice?
- If yes, could you please explain by giving us a few examples of the cost accounting models used by different departments in a facility or types of models based on the size of the facility?

1. How would you describe the current state of cost accounting in India in terms of the approaches being used for cost assessment across different provider organizations? In your opinion, which method or system of cost accounting can be the best practice in the Indian context and why?

(Hints: regulation, systems, implementation)

1. What do you think the motivating factors are for using (explicit) management accounting/cost accounting systems (for example patient level cost analysis) in the hospital sector in India (probe: if regulatory only ask about other forms of cost accounting)
2. How do you think a (CEO of a) hospital/provider organization think they might benefit from an explicit cost accounting system (do you think this would be different in different provider organizations in India)?
3. Have you seen any changes in cost reporting practice recently? What form have these changes taken?
4. In many countries with national health insurance programs, empanelled providers are required to submit cost information to the central authority to inform reimbursement rates. Do you think that different provider organizations will be open to reporting cost accounting information to NHA/SHA via a standardized platform for the assessment of hospital healthcare costs? Why?
5. Can you comment on the feasibility of such a system given the organization of healthcare delivery in India? Can you explain why and if this might vary across different provider organizations?

(Hint: Will it depend on the:

- The type of hospital (private/public/empaneled/PPP)
- Level of care/facility provided
- Multiple insurance providers
- Any existing state norms
- Health packages provided
- Capacity/transparency
- Others?

1. Do you think that cost information is currently being used in the public as well as the private sector to inform prices/charges?

If yes, can you describe examples of set-up where-in it is in practice in the public sector and comment on whether it has been useful or not? Also, can you briefly describe the cost accounting mechanisms used in these set-ups?

1. Can you reflect on how the charges in the hospital industry are set for different payers (i.e. individual patients, private insurers, publicly funded insurers)? And how does this vary across provider organizations e.g. private for profit, not for-profit, and public sectors/empanelled vs non/ rural vs urban/ smaller vs larger or networks?

- Administrative pricing
- Negotiations between payers (insurance companies/government) and provider organizations.
- Existing price in the market (market dynamics)/competitive pricing
- Any role of the general practitioner/doctor
- Others?

| [**DEFINITIONS**](https://www.brookingshealth.org/why-brookings-health/health-care-value/understanding-medical-prices/charge-cost-price)  **Charge:**  A charge is an amount a healthcare provider organization sets for services rendered before negotiating any discounts. The charge can be different from the amount paid.  **Cost:**  Varies by the party incurring the expense.  **To the patient**, the cost is the amount payable out-of-pocket for healthcare services. This may include deductibles, co-payments, co-insurance, amounts payable by the patients for services that are not included in the patient’s benefit design, and amounts balance-billed by out-of-network providers. Health insurance premiums constitute a separate category of healthcare costs for patients, independent of healthcare service use.  **To the health care provider**, the cost is the expense (direct and indirect) incurred to deliver health care services to patients.  **To the insurer**, the cost is the amount payable to the health care provider (or reimbursable to the patient) for services rendered.  **To the employer**, the cost is the expense related to providing health benefits (premiums or claims paid).  **Price:**  Price is the total amount a healthcare provider expects to be paid by payers and patients for healthcare services. |
| --- |

1. Do you see a role in cost accounting information in the setting of reimbursement rates for PMJAY?

1. How do you think we can make cost accounting a standard practice given the organization of healthcare in India and what could be the way forward?

**********
