# Supplementary material for: Health care cost accounting in the Indian hospital sector
Source: Health Policy Plan. 2024 May 30;39(7):731–40. doi: 10.1093/heapol/czae040 (PMC11308608; doi:10.1093/heapol/czae040)
Supplement: czae040_Supp [file czae040_supp.zip › suppl_data/Table of Quotes.docx]

| **Table 2: Summary of the Thematic Analysis** |
| --- |

| **THEMATIC CODES** | **QUOTES** |
| --- | --- |
| **Status of Healthcare cost accounting** | *“The hospital industry is not very mature in terms of costing system”.*  *“It's only the big players who really have done costing, and the 80% of health care is provided by the medium-sized? players who have absolutely no idea what is the costing ………. they don't know costing.”*  *“In the past there has not been much conscious attempt in the healthcare industry to, uh, focus on the costing systems. …….…. There was not a structured framework”.*  *“There is no such practice of cost accounting in the public sector ……… cost accounting becomes crucial when you need to set prices. And, so the public sector is not bothered about how much it is costing at the management level.”*  *“…. every institution, including us when we started, we did not start with the cost accountant, because they are not available. And then you do not want to do that [costing] exercise, because there is already a competitive pricing which is existing [and is used to set prices for delivering healthcare]. And then you will tend to just work with these [competitive] prices as costing is a costlier exercise, which is not done.”*  *Now they're trying to do studies on their own. Even that awareness is, uh, catching up. Probably if I, if we are able to develop a good tool across the country and publish certain standards. Unfortunately, costing institute standards are like kind of frozen now”* |
| **Legal and regulatory requirements around cost reporting in India** | *“There is a regulatory requirement but it is weak, I mean there is no stipulation that your pricing should be linked to costing. I mean we are supposed to engage a cost accountant to give us a certificate every year that we have a cost accounting system in the organization. That is the only regulation that we have.”*  *“Public sector never comes under regulations. No rules or law applies to the public sector. So, there is no level playing field.”* |
| **Motivation to have cost accounting systems in hospitals** | “*I want to look at it because I want to know the profitability, and pricing of the procedure and when I know my profits then only, I can decide how can I share it with my doctors.”*  *“How do we really look into the competitiveness of that area, whether the rates are competitive, whether my efficiency is competitive, whether my procedures are competitive? The hospitals have now started looking into that.”*  *“The general perception is that the reimbursement prices are very low. I don't know. We have never tried to analyze it that way, that how low are they? But I am sure if a proper costing study is done and some reasonable pricing is offered, people like me who are scared to go for such schemes because I think they're a loss, they'll be encouraged to take up such schemes.”*  *“We now understand that digital is the new frontier. We have new IIT [Indian Institute of Technology], IIM [Indian Institute of Management] personnel for digital transformations and tools like Power BI those will help us to see even the costs incurred at the doctor’s [human resource] level.”* |
| **Feasibility/willingness for collating and reporting cost information** | *“If the tool is developed, yes, the private sector will be too willing to happily do that”.*  *“I think by now we are able to get most data from the system…[for] something like patient food…………. those things you have to manually add to it and consolidate in your direct cost…. it's not that much of an effort. In fact, if we have to invest in the system that is more detailed and will require more investment, which we would rather, you know, spend on medical equipment, et cetera”*  *“Secondly, I have spoken to enough number of providers over years, and they always eventually come to that, you know, uh, why should there be a conversation about margins? At the moment, price is available to me, if the cost becomes available to me, then the margin is visible to me. Yeah. And it becomes a trade margin-oriented discussion which every provider wants to avoid.”*  *“I think you can ask for volunteers who are willing ……. You can start with that, that will give you enough sample size………. If that is not successful, maybe then you can, as legislation put that, you know, every, any 200 hospitals will pick up and they have to provide.”*  *“I [am] willing to give my financial data …[in], whatever format is the, will be provided to me.”*  *“It’s very difficult [to standardize cost accounting practices] for the simple reason, because in India I don't think different smaller setups have, or even bigger setups have any standard set of protocols. Each setup has their own protocols and its own standard.”* |
| **Challenges to setting up efficient cost accounting systems in hospitals** | *“NHA who is looking after the PMJAY scheme, uh, they directly didn't become part of the [cost accounting] study [aimed at assessing the cost of healthcare]. Uh, so there, here was that whatever exercise we may do, it may not become acceptable to NHA.”*  *“[Even while collecting information on healthcare costs] Then you don't know whether to trust that data or not because it's voluntary data. So, we try to do it at a hospital association level. So, the problem starts with there then everyone can question the validity of that data and therefore, you know, it's, It's a chicken and egg.”*  *“The major challenge is not only in developing the tool but how do you integrate it with the financial accounting. So, from billing, how do you segregate? And then paste them into my ledger accounting.”*  *“so there's a challenge, many of the IT systems are not oriented towards the cost. Of course, today HIS [health information system] IT [Information & Technology] system available in the market is standard, good and robust, but there is a problem in implementing it.*  *“Most of the Hospital Management Information Systems for the financial modules most of the people don't incorporate this cost accounting modules. As I mentioned, to design a module that caters to the complexity of the usage pattern, the cost patterns, and the allocation patterns. So, it is difficult, normally we try to build our own IT systems to adapt to these complexities. So, there is no off-the-shelf cost accounting systems which can cater to our requirements.”*  *“Doctors don't understand the impact of costing. Most of them, they do know that we have to be cost-effective, but when it comes to sharing relevant data in working with the cost accounting professionals or people like me, the doctors think that they know, so that's a problem. There is a reluctance to share the data. If you know that the current cost of my service, you will, you know, not allow me to make a decent return.”* |
| **Using cost information for price setting** | *“If relevant, it would be there in tertiary care settings, not in the primary or secondary care settings. There is no capacity.”*  *“There is no need also in the public system and their orientation, the entire emphasis is different”*  “*So that is all the pricing mechanism. Apollo fixed at 100 Rs. I will fix it at 65 Rs. Okay. My Star Health insurance [reimbursement] is at 100 Rs. My Gypsa [reimbursement] is about 80 rupees. My, uh, CGHS [reimbursement] should be not less than at least 65 today. So pricing is not related to costing. It is market driven.*”  *“So, it is more of a competitive pricing when we come to set pricing in, um, healthcare industry and private sector. But then when it comes to the government, it actually sees the cost of delivering healthcare in different set-ups maybe private, government, and then different tiers of cities and everything.”*  *“The other very rough method of, you know, deciding what we should be charging is by analysing what our neighbours are charging. If, if I know my neighbor is charging this much……. then it depends on the kind of policy that I want to adopt. Suppose I want to….. be looked upon a center with quality……. probably I'll keep my charges a little higher than those, or suppose I want to, you know, work with volumes, then I would like to attract by charging less. That's kind of a policy which we adopted, and I can tell you most of these smaller setups are adopting.”*  *“There are two, uh, two different aspects of looking at that. One aspect is where the government is funding the, or any insurance authorities funding that they necessarily will look into the cost aspect. Also, they will not approve the rates without looking to the cost of those services and all that. So like this NHA or the various, uh, insurance authorities, they look into the cost, but as for the non-structured, private person has to pay for the cost of their health, uh, for that, why should the hospital apply cost? They will apply the costing for their own decision-making process, and that they will not do the structured costing. They will do the thumb rule costing, what you call the rough costing system from experience, I know that this, this activity will cost me so much of rupees.”* |
| **Recommendations to improve the state of cost accounting** | *“If we really want to spread the knowledge of costing in the healthcare industry and improve that system, we have to have series of workshops and seminars across the country wherein we should involve the senior people from the government also”;*  *“The NHA should handhold providers to drive their efficiency in the system by providing them training in costing and based on costing how they should try to make it still viable in the package that you have defined.”*  *“Most of the small to medium hospitals sized hospitals cannot invest into the teams which can undertake this detail. We need to have robust … management systems and … …. internal audit systems [that monitor] their consumption pattern.”*  *“The government may be advised to set up such kind of regulatory guidelines [for smaller and medium sized hospitals similar to] corporate hospitals, which is there. To the non-corporate …… they can definitely extend those guidelines of mandatory maintaining the cost reports and all that, so that way automatically the system will improve.”*  *“So, there is a, there is a, uh, now a role of the cost accountants in this…...”*  *“A certain format should be provided to each hospital and they can be asked to give you the general finances. Or some private body, uh, which is expert in costing, can be handed over, uh, uh, to study the costs incurred in various kind of small size midsize and large hospitals. I'm sure that's the best way to, uh, you know, get an average of what will be the cost incurred by different procedures.”*  *“Absolutely, so, maybe you could develop a repository. Everybody would know what is the rate from each hospital. Many hospitals would be willing to participate and they could be the consortium with they can bring their own rate.”*  *“So however, confidential you would like to keep, and then at least come up with some kind of a minimum standard requirement.”* |
| **Previous Costing initiatives** | *“Took an initiative that why not we undertake and complete that exercise of undertaking the cost of medical procedures in various hospitals in the country and all that. We selected some 70-72 hospitals of different sizes from across the country from north, east, south, and west. We were quite advanced in that exercise. We prepared a lot of templates for collecting the data from various hospitals”; “Subsequently, FICCI did a study and AIIMS as well. Their methodology was also different and we did another study. But not surprisingly, all three studies had same results/conclusion”.* |
| **Relationship between the Government & healthcare providers** | *“….. they don't want to share it with the government and NHA…”.*  *“The costing is a very sensitive, uh, data. No corporate entity is willing to share the, the cost data with, uh, outside the company, even within the organization. They don't want to share within one department or another department, until we have a structured, regulatory framework, the hospital industry is not going to share any type of information easily with you. They are not willing to share.”*  *“We don't get any benefit from government. We've not got land, you know, we pay 30% duty on medical equipment that we import…………… we don't get any subsidy like IT industry gets [like the] tax subsidy for initial five years…. So, we don't get any benefits. So why should we do it? You know, there's no reason for us to do it.”*  *“the Ministry of Health had set up the committee and put 2 or 3 of us across on the committee. They did not …. involved us in anything, and then came up with their own results which nobody …. actually tested”.*  *“the private players have to be in the process of costing, and unfortunately it [now] looks non transparent and we don't know how [the NHA] does [costing] for each [service] one, the but cost [reimbursement rates] just comes in to us and so the participation becomes difficult and challenging; the Private Player should be included in the dialogue.”*  *“We have to work out a system where the private sector is included in a fair manner. More than costing I mean I would invest in building trust and relationships, then everything becomes easier”*  *“I think we're trying to ensure that the government and the private place work together, because if you are working together and we see each other’s’ points of view, I think it would be much easier and ultimately the person who is going to benefit ……. is the patient”;*  *“Government needs to look at private healthcare as a partner rather than adversary”* |
| **Views on AB PM-JAY pricing** | “*I need at least 15,000 Rs. and you are giving me 8,000 Rs. So why do I, you know, share my data with you?”*  *“Ayushman Bharat, when you try to pitch in and then they were giving us, per caesarean delivery, Rs. 8,000. My doctor fee in tier two cities itself is about 3,000 to 4,000 Rs. minimum and an experienced 15 years gynaecologist will charge at least about Rs. 5,000 per surgery. My consumables are at least Rs. 2,500 to 3,000. So, all put together, it's about 8,500 rupees. Then the rest of it. Even a general ward is for 600 Rs. per day, and I will need for at least for five days, assuming the advent of infection, bleeding, postpartum complications, and all that. So, I'm thinking about four to five days, four days of stay and then my cost is at least around 12,000. My margin another 3000- 4,000 Rs., I need at least 15,000 Rs. and you are giving me 8,000 Rs. So why do I share my data with you?*  *“Should the government be setting its prices based on the costs of those services, or should it be also doing market surveys to look at what the going rates are?”*  *“Government reimbursement rates are very low and the reimbursement processes are very slow.”* |
| **Platforms used by providers for cost accounting** | *“Power BI is data visualization tool and not a cost accounting or costing tool. Yeah, but for visualization also it will be doing all the calculations in the backend. In the backend also we have Microsoft excel.*  *“Yeah, so we, um, the two key, um, applications we use, one is hospital information system, which is often the billing system. Uh, and we have then enterprise application like SAP or, or where we have all our inventories, um, financial accounting data. So the combination of both these systems, we, uh, do all our, you know, um, financials*. “ |
