# Supplementary figures and images for: Health care cost accounting in the Indian hospital sector
Source: Health Policy Plan. 2024 May 30;39(7):731–40. doi: 10.1093/heapol/czae040 (PMC11308608; doi:10.1093/heapol/czae040)

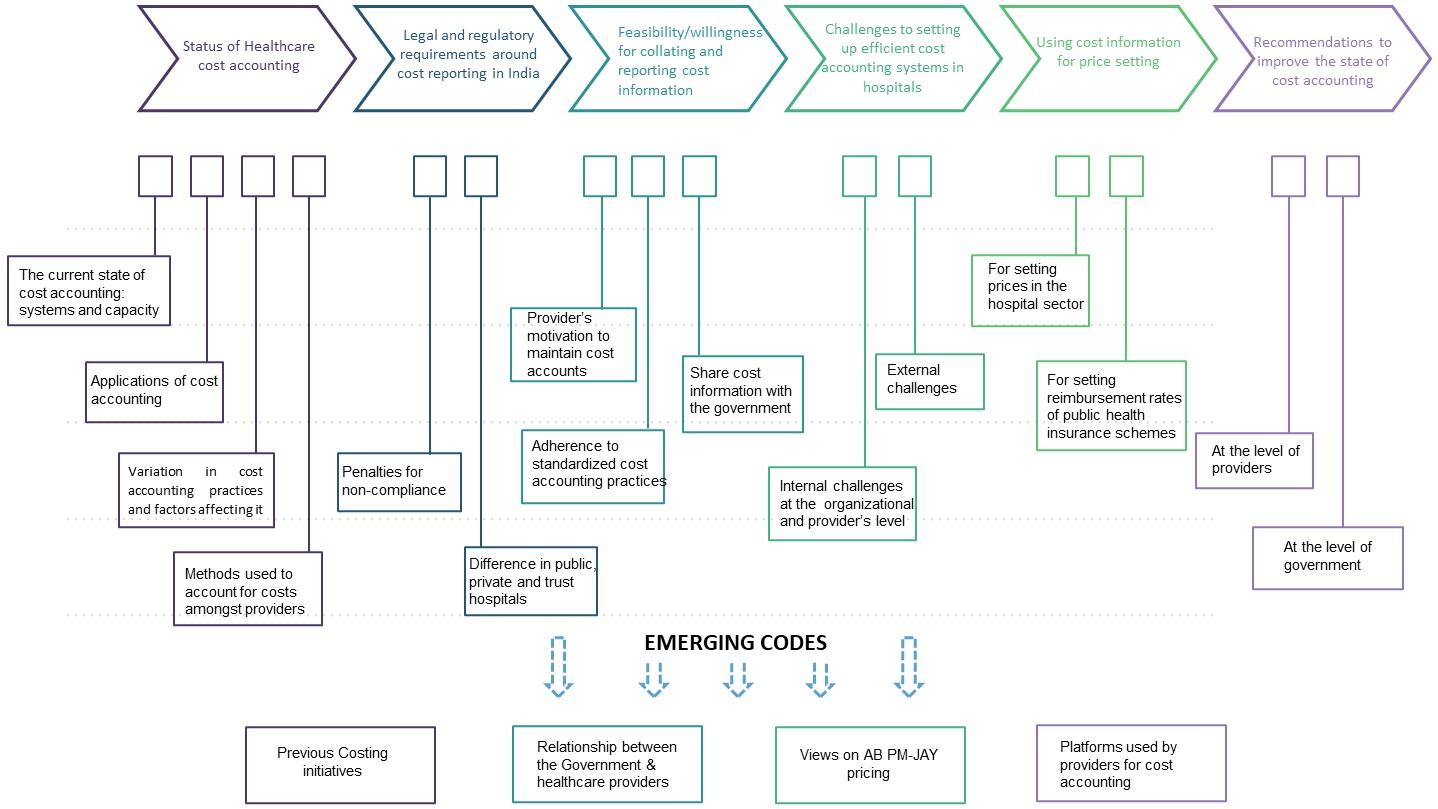

Supplement: czae040_Supp [file czae040_supp.zip › suppl_data/Cost accounting_Figure 1.jpg]

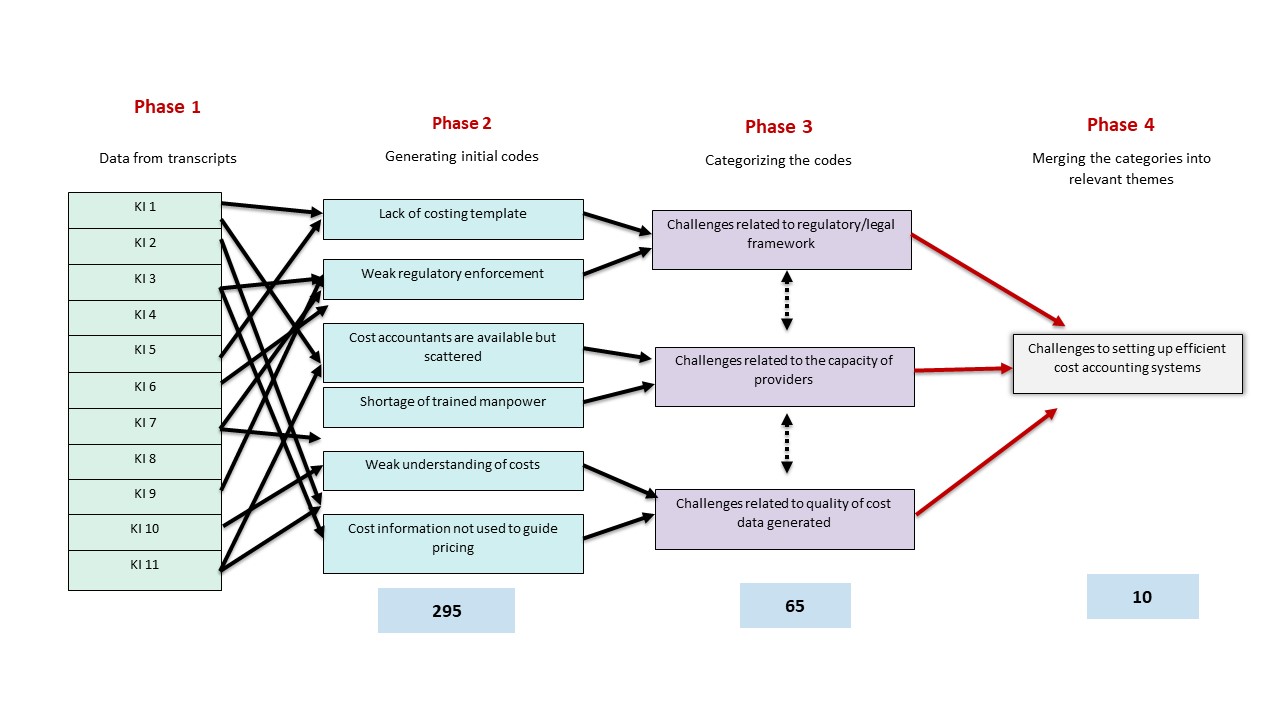

Supplement: czae040_Supp [file czae040_supp.zip › suppl_data/Figure 2 Coding Framework.jpg]

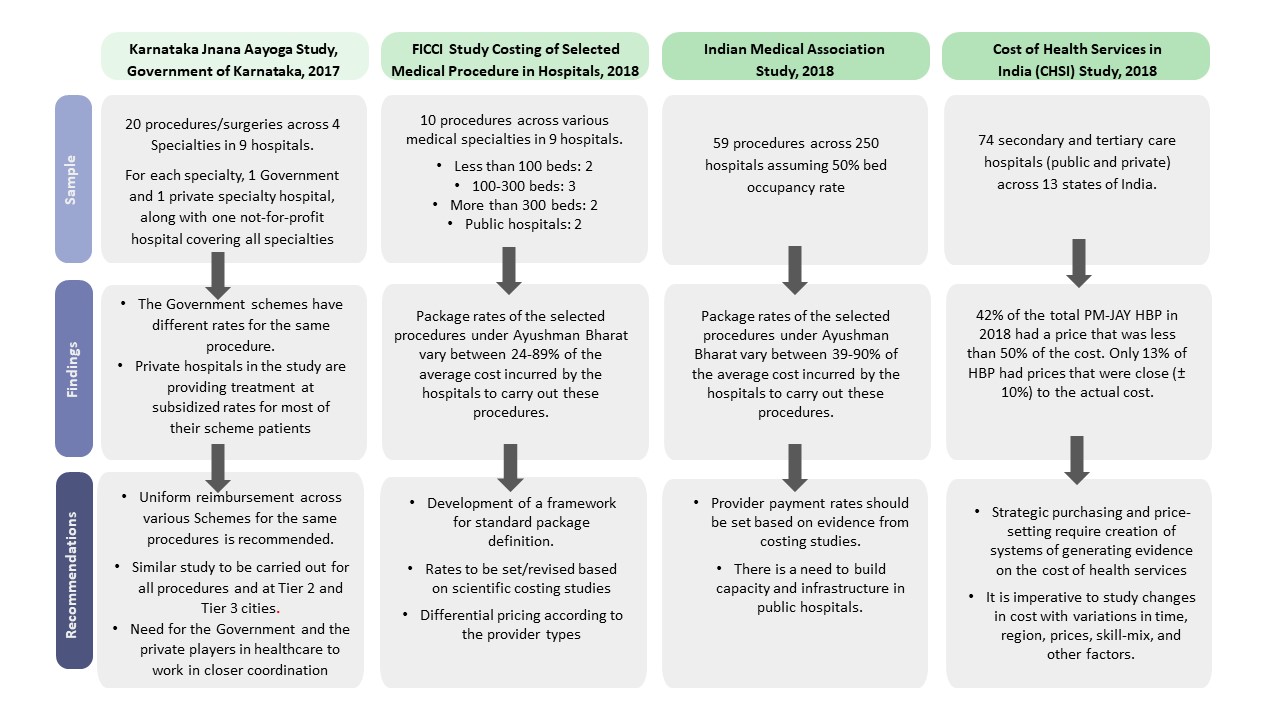

Supplement: czae040_Supp [file czae040_supp.zip › suppl_data/Figure 3.jpg]
